# Perturbing mitosis for anti‐cancer therapy: is cell death the only answer?

**DOI:** 10.15252/embr.201745440

**Published:** 2018-02-19

**Authors:** Manuel Haschka, Gerlinde Karbon, Luca L Fava, Andreas Villunger

**Affiliations:** ^1^ Division of Developmental Immunology, Biocenter Medical University of Innsbruck Innsbruck Austria; ^2^ Centre for Integrative Biology (CIBIO) University of Trento Povo Italy

**Keywords:** apoptosis, BCL2 family, mitotic arrest, p53, slippage, Autophagy & Cell Death, Cancer, Cell Cycle

## Abstract

Interfering with mitosis for cancer treatment is an old concept that has proven highly successful in the clinics. Microtubule poisons are used to treat patients with different types of blood or solid cancer since more than 20 years, but how these drugs achieve clinical response is still unclear. Arresting cells in mitosis can promote their demise, at least in a petri dish. Yet, at the molecular level, this type of cell death is poorly defined and cancer cells often find ways to escape. The signaling pathways activated can lead to mitotic slippage, cell death, or senescence. Therefore, any attempt to unravel the mechanistic action of microtubule poisons will have to investigate aspects of cell cycle control, cell death initiation in mitosis and after slippage, at single‐cell resolution. Here, we discuss possible mechanisms and signaling pathways controlling cell death in mitosis or after escape from mitotic arrest, as well as secondary consequences of mitotic errors, particularly sterile inflammation, and finally address the question how clinical efficacy of anti‐mitotic drugs may come about and could be improved.

**Glossary**
APC/C: anaphase‐promoting complex/cyclosome
BAD: BCL2‐associated agonist of cell death
BAK1: BCL2 antagonist 1
BAX: BCL2‐associated X
BCL2: B‐cell CLL/lymphoma 2
BCLX: BCL2‐like 1
BH3: BCL2 homology domain 3
BID: BH3‐interacting domain death agonist
BIM: BCL2‐interacting mediator of cell death
BMF: BCL2‐modifying factor
BUB1: budding uninhibited by benzimidazoles 1
CDC20: cell division cycle 20
CDK1: cyclin‐dependent kinase 1
CENP‐E: centromere protein E
cGAS: cyclic GMP‐AMP synthase
CIN: chromosomal instability
IRF3: interferon regulatory factor 3
KIF11: kinesin family member 11
MAD1/2: mitotic arrest deficient 1/2
MCC: mitotic checkpoint complex
MCL1: myeloid cell leukemia sequence 1
MDM2: mouse double minute 2, human homolog of
MOMP: mitochondrial outer membrane permeabilization
MPS1: monopolar spindle 1 kinase
NF‐κB: nuclear factor kappa B
PIDD1: p53‐induced protein with a death domain 1
PLK1: polo‐like kinase 1
PTM: post‐translational modification
PUMA: p53‐upregulated mediator of apoptosis
SAC: spindle assembly checkpoint
STING: stimulator of interferon genes
TNF: tumor necrosis factor

## Introduction

Anti‐mitotic drugs, including vinca alkaloids or taxanes and derivatives that target microtubules, are used successfully in the clinics to treat multiple types of cancer but negative side effects, such as neurotoxicity and frequent drug resistance, limit their therapeutic use 1. This has led to the development of second‐generation compounds, often referred to as “mitotic blockers”, that aim to interfere with proper spindle formation, chromosome segregation, and/or mitotic exit, for example, by targeting microtubule motor proteins, including kinesins KIF11 or CENP‐E, polo‐like kinase (PLK)1, or Aurora A kinase, as well as the APC/C E3‐ligase complex, respectively. However, these targeted drugs had overall limited success in clinical trials so far and proved often less effective as before‐mentioned microtubule‐damaging agents 2. Along similar lines, another class of compounds, referred to as “mitotic drivers”, triggering premature mitotic exit by targeting key mitotic kinases such as MPS1 or Aurora B, forcing chromosome missegregation or mitotic slippage with cytokinesis failure, respectively, are under development for cancer treatment 2, 3. All these compounds can trigger cell death, the preferred outcome in clinical use 4, 5, but also numerical as well as structural chromosomal instability (CIN) and/or tetraploidy‐mediated aneuploidy in surviving cells, hallmarks of cancer evolution, and drivers of drug resistance 6.

What all these compounds, spindle poisons, mitotic blockers, and drivers, do have in common is that they can trigger a p53 response in non‐transformed cells, as well as in tumors carrying wild‐type p53. This response is still poorly understood mechanistically, but clearly distinct from the one induced by classical triggers of DNA damage, such as ionizing radiation or conventional chemotherapy 7, 8. In addition, many (if not all) of these drugs can elicit apoptotic cell death, at least in tissue culture 9, 10, 11. This type of cell death may or may not engage p53, and the basis of cell death paradigms triggered upon erroneous mitosis is considered to be very heterogeneous and molecularly ill‐defined, hence often summarized as “mitotic catastrophe” 5, 12. The feature of promoting CIN and aneuploidy upon drug treatment appears as a double‐edged sword: On the one hand, aneuploidy itself will have a negative impact on the cellular fitness, but on the other hand, it harbors the inherent problem that therapy exploiting these features will be selecting for complex karyotypes that promote cell survival and aneuploidy tolerance. This is drastically facilitated by p53 loss, leading to more aggressive disease, posing a major challenge for safe clinical application 6, 13, 14.

Moreover, recent data actually suggest that doses of anti‐mitotic drugs needed to induce apoptosis of cancer cells *in vitro* are likely not achieved *in vivo* and, hence, do not efficiently arrest cells in mitosis. For example, paclitaxel rather causes multipolar cell divisions ultimately limiting cancer cell fitness 15. Ultimately, the clinical efficacy of these drugs may actually also rely on the induction of inflammatory responses in cells experiencing DNA damage upon mitotic errors, either before or upon their, potentially immunogenic, death 15, 16, 17. As the type of cell death induced by these and other anti‐cancer compounds clearly impacts on anti‐cancer immunity, sterile inflammation elicited by anti‐mitotic drugs most likely constitutes a highly underappreciated and underexplored feature contributing substantially to their clinical efficacy 17, 18.

With this review, we are aiming to highlight most recent developments identifying the molecular machinery that is engaged to execute cell death upon stalled mitosis or upon mitotic exit, with a focus on the BCL2 protein family and their regulation in and out of mitosis. This overview will be complemented with a shorter summary on recent studies identifying novel players leading to p53 activation upon extended mitotic arrest, or in response to supernumerary centrosomes, that have been highlighted recently nicely elsewhere 19, 20. Ultimately, we would also like to end with more thoughts on the cross talk between the mitotic machinery with the one controlling cell death and potential links to sterile inflammation as the latter clearly impacts on the clinical efficacy of drugs aiming to target cancer cells by manipulating mitotic fidelity.

## Molecular control of mitotic arrest

**Figure 1:**
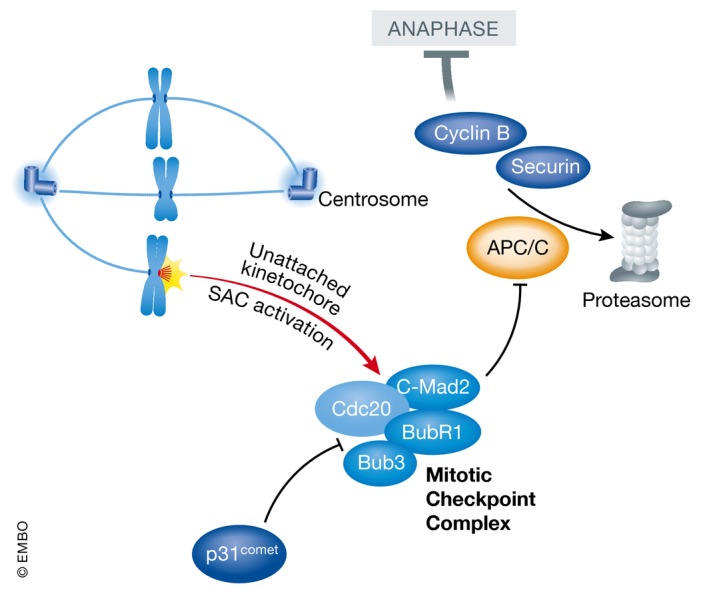
Anti‐mitotic drugs activate the spindle assembly checkpoint (SAC) Unattached kinetochores trigger the activation of the SAC, leading to inhibition of prometaphase to anaphase transition and mitosis by blocking the activity of the APC/C E3 ligase complex. The mitotic checkpoint complex (MCC) thereby inhibits CDC20 from aiding substrate recognition by the APC/C (e.g., cyclin B or securin, degraded for mitotic exit), thereby enhancing mitotic arrest. MCC function can be antagonized by p31^comet^ that can drive mitotic exit but seemingly also exerts alternative anti‐apoptotic functions in cells arrested in mitosis.

Mitotic arrest is implemented by the spindle assembly checkpoint (SAC) machinery which enables and monitors proper segregation of sister chromatids to daughter cells during mitosis. Chromosome segregation requires the mitotic spindle apparatus, whose tubulin fibers originate from two microtubule‐organizing centers that, in metazoans, are composed by centrosomes, positioned at opposing sides of the cell during mitosis. Tubulin fibers attach to special proteinaceous structures, called kinetochores, that assemble on centromeric chromosome regions. The SAC acts as a surveillance mechanism to ensure that the onset of anaphase involving the removal of sister chromatid cohesion and separation only occurs when all chromosomes are attached to the mitotic spindle in the correct configuration, that is known as bi‐orientation (Fig 1) 21. As long as a single chromosome is not bi‐oriented, the key effector of the SAC, the so‐called mitotic checkpoint complex (MCC), keeps the cells arrested in prometaphase. This is achieved by the inhibition of the large E3 ubiquitin ligase complex called the anaphase‐promoting complex or cyclosome (APC/C). The APC/C, once relieved by the inhibition of the SAC, ubiquitinates two key substrates, namely cyclin B and securin, for proteasomal degradation allowing separase to cleave cohesin for chromosome segregation and anaphase progression (Fig 1). The APC/C requires a particular cofactor, CDC20, for degradation of cyclin B and securin in mitosis. This is in turn the target of the control exerted by the SAC on the APC/C 21.

On kinetochores lacking microtubule attachment, the actions of Aurora B kinase lead to the recruitment of monopolar spindle (MPS)1 kinase to the kinetochore 22. MPS1 kinase localization at kinetochores relies on binding to members of the outer kinetochore NDC80 complex 23 and is mutually exclusive with microtubule binding 24, 25. MPS1 activity is in turn responsible for the recruitment of several other key checkpoint components such as the MAD1:MAD2, BUB3:BUB1, and BUB3:BUBR1 complexes 21. How unattached kinetochores that become decorated by checkpoint components can act as catalytic platform for the generation of a diffusible inhibitor effectively targeting the APC/C throughout the whole cell has been a matter of intense investigations during the past 15 years. Central to our understanding of this phenomenon is the so‐called MAD2 template model 26, relying on the observation that the SAC component MAD2 has two natively folded states, open O‐MAD2 and closed C‐MAD2 27. O‐MAD2 is the unoccupied state where MAD2 may receive one of two binding partners, MAD1 or CDC20. The closed conformation is adopted when one of those two proteins is bound by MAD2 28. Critically, the protein can form asymmetric homodimers 29 in which a kinetochore associated and MAD1‐bound C‐MAD2 can recruit a second moiety of MAD2 in the open conformation 30, 31. The MAD1:C‐MAD2 template can catalyze the conformational change of the O‐MAD2 moiety, thereby facilitating the formation of a C‐MAD2:CDC20 complex, that is then able to associate with BUB3/BUBR1, leading to a fully functional APC/C inhibitor, the MCC 32, 33.

Intriguingly, while sequestration of CDC20 by the MCC plays a role in inhibiting the APC/C, the MCC‐CDC20 complex can actually bind to the APC/C 34. There are three main modes of actions currently proposed to explain how the MCC‐CDC20 inhibits the action of the APC/C: (i) BubR1 was shown to act as pseudosubstrate for the APC/C without being degraded 35; (ii) the MCC‐CDC20 binds to the APC/C in a way that CDC20 is delocalized from its proper binding site and this then prevents the APC/C from recognizing its substrates 36; and (iii) the APC/C‐bound MCC can bind to free CDC20, negatively influencing its ability to activate the APC/C 37. Regardless of the mechanistic details, CDC20, a co‐activator of the APC/C when devoid of SAC proteins, becomes an APC/C inhibitor when bound to SAC proteins within the MCC.

After proper bipolar attachment of spindle microtubules to the kinetochores of all sister chromatids is established, the SAC is turned off so that the cell can progress in anaphase. There are several mechanisms that concur to effectively shut down the SAC: (i) The MAD1‐C‐MAD2 complex is stripped away from the kinetochores in a dynein‐dependent fashion, therefore terminating the generation of new MCC complexes 38; (ii) microtubule binding to the NDC80 complex dissociates MPS1 kinase from kinetochores, thereby inhibiting critical phosphorylation events that are required for the recruitment of SAC components to the kinetochore 24, 25; (iii) an important brake of the SAC is p31^comet^ which binds to the dimerization motif of C‐MAD2 39, 40, which appears to play a major role in the disassembly of the MCC 41 . While p31^comet^ acts even during SAC signaling to regulate checkpoint homeostasis 42, the regulation of p31^comet^ activity when the SAC has to be silenced remains less clear; and (iv) finally, the actions of the phosphatases PP1 43, 44 and PP2A 45 at the kinetochore are required as well to efficiently terminate the SAC.

## Anti‐mitotic drugs and their potential biological effects

Anti‐mitotic drugs such as vinca alkaloids and taxanes interfere with microtubule dynamics and therefore prevent the mitotic spindle from attaching amphitelically to kinetochores 46. This chronically activates the SAC, blocking the cells from progressing through mitosis. The EG5 kinesin inhibitors on the other hand prevent the segregation of the two centrosomes leading to a monopolar spindle and chronic SAC activation 47. However, two aspects have to be kept in mind to explain how cells can escape mitotic arrest before undergoing cell death. First, even when the SAC is maximally active, cyclin B is slowly degraded 48. Once cyclin B levels drop beneath a critical threshold, the cell can exit mitosis, here, often into a polyploid interphase. This is referred to as mitotic slippage or mitotic checkpoint adaption 48. The second issue is that while one unattached kinetochore is indeed sufficient to activate the SAC, the SAC does not work in a bimodal fashion but can exert varying signaling strength 49. Hence, different mitotic inhibitors can activate the SAC to a different extent 49. It therefore follows that intrinsic factors such as the levels of MAD2 49 or p31^comet^ 50 influence the strength of the SAC and, by extension, the speed and rate of mitotic slippage without fully abolishing the actions of the SAC.

Intriguingly, different cell types studied for the long‐term consequences of mitotic arrest by live‐cell imaging often show a strikingly different behavior, that is, mitotic cell death vs. slippage and mitotic exit, preventing death in mitosis. Seminal work by the Taylor laboratory has led them to propose the competing network hypothesis that accounts for the differential behavior of cells in this setting 9, 12. In short, the idea behind the model is that not only the molecular machinery controlling cell division but also the apoptotic machinery is activated in mitosis and that mitotic arrest is intrinsically pro‐apoptotic. Hence, when a cell gets trapped in mitosis, an apoptotic activity builds up, ultimately leading to activation of proteases of the caspase family and cell death, while on the other hand, mitotic exit allows escape from death, at least in the next G1‐phase. As such, the gradual degradation of cyclin B by APC/C activity sets the time to mitotic exit that can differ between cell types but also between individual cells of the same cell line in a single experiment, generating often heterogeneous response patterns *in vitro*. When cells experience a drop of cyclin B levels below a critical threshold before mitochondrial integrity is perturbed, cells exit mitosis and reset the apoptotic clock back to baseline 9, 12, 51. Not surprisingly, a series of cell death proteins are defined substrates of mitotic kinases and become heavily modified in mitosis but are then quickly de‐phosphorylated again upon mitotic exit (see below). In support of the competing network model, overexpression of anti‐apoptotic proteins, such as B‐cell CLL/lymphoma 2 (BCL2), can prevent death of mitotically arrested cells long enough for cyclin B levels to drop below a threshold for slippage. Similarly, deletion or inhibition of key apoptotic effectors, including the BCL2 family members BAX/BAK1, or chemical inhibition of caspases induces similar effects 5, 9, 11, 52, 53. On the other hand, preventing mitotic exit, for example, by depletion/deletion of CDC20, pharmacologic inhibition of the APC/C, or overexpression of (non‐degradable) cyclin B, and arresting cells in M‐phase with high efficiency ultimately resul in apoptosis 9, 53, 54, 55, 56. Extended mitotic arrest is also associated with increased autophagy rates that even seems to contribute to death to some degree and a metabolic switch to enhanced glycolysis for survival, but eventually, all cells die. This death is characterized by necrosis‐like morphology when cells are unable to trigger apoptosis or exit mitosis in due time 53.

## P53 activation in response to mitotic errors

**Figure 2:**
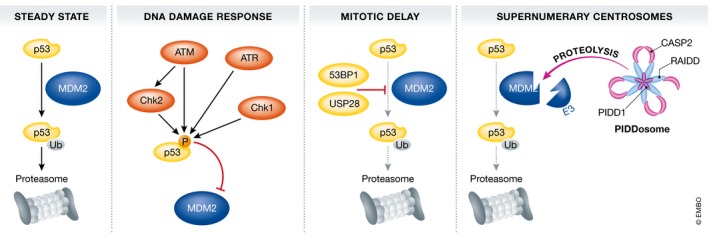
Multiple independent pathways can lead to p53 stabilization In steady state, cytoplasmic p53 levels are kept low by continuous MDM2‐mediated ubiquitination and proteasomal degradation. Within the canonical DNA damage response, p53/MDM2 interactions are neutralized by phosphorylation of p53, abrogating MDM2 binding, that are executed by DDR kinases ATM, ATR, CHK2, and CHK1, depending on the type of DNA damage encountered. Cells spending an extended period of time in mitosis are also able to activate p53 for subsequent cell cycle arrest in the next G1‐phase. There, MDM2 activity is antagonized by the activity of a de‐ubiquitinating enzyme, USP28, all held together by the p53‐binding protein 53BP1. Finally, upon cytokinesis failure, extra centrosomes present in such cells activate the PIDDosome multiprotein complex, comprised out of the p53‐induced protein with a death domain (PIDD)1, a linker protein called RAIDD and a protease of the caspase family, that is, caspase‐2. Upon activation in the PIDDosome, caspase‐2 can process MDM2, removes its E3 ligase domain, and thereby promotes p53 stabilization.

Interestingly, delaying mitotic exit beyond a given threshold in time (about 90 min in RPE1 cells 8) can promote a p53 response in mitosis that is “carried over” into the next G1‐phase 8. This response involves p53‐binding protein 1 (53BP1) and the de‐ubiquitinating enzyme USP28 that counteracts MDM2, thereby promoting stabilization of p53 promoting cell cycle arrest in the next G1‐phase 57, 58, 59. In addition to extended mitotic duration itself, chromosome congression and segregation defects can also result in p53 stabilization 60, 61. Yet, whether the quantitative measurement of mitotic timing and sensing the fidelity of chromosome segregation are mechanistically coupled or independent pathways remains to be clarified. Irrespective of the activating mechanism, whether the p53 response to mitotic abnormalities initiates already in mitosis and contributes to cell death induced by microtubule‐damaging agents remains unclear at present. Given the postulated absence of transcription in mitotically arrested cells, this appears unlikely. However, this dogma has been challenged lately 62. Moreover, albeit not generally accepted, non‐canonical direct cell death‐promoting functions of p53 have been reported 63. Certainly, this p53 activity can impact on cell fate in the next G1‐phase when transcription is reinitiated, as several BCL2 family proteins, such as BAX, PUMA, or NOXA, are confirmed p53 targets 63. It remains possible that these genes are bookmarked by p53 already in mitosis for low‐level transcription and swift expression upon mitotic exit 62, defining post‐mitotic cell fate (Fig 2).

Remarkably, however, in some settings, discussed in more detail below, increasing apoptotic thresholds in cells arrested in mitosis only delays cell death without resulting in increased mitotic slippage, suggesting that both thresholds are affected simultaneously. Moreover, caspase activation has been noted to repeatedly occur at low level in mitotic cells, leading to the activation of caspase‐activate DNAase and DNA damage that needs to be corrected after mitotic exit as otherwise fostering genomic instability 7. Finally, it has to be pointed out here that key apoptotic effectors, such as caspase‐3 or caspase‐7, have been repeatedly linked to impaired proliferation or migration of several cell types in the blood, for example, by disabling p21 64 or p27, by proteolysis 65. Hence, not all caspase activation noted in mitosis must necessarily lead to apoptosis or genomic instability, but may ultimately contribute to proliferation or migration control by targeted proteolysis of selected substrates. Along similar lines, overexpression of BCL2 or loss of pro‐apoptotic effectors, such as BIM, has been linked to impaired proliferation of lymphocytes upon mitogen stimulation 66. Whether this phenomenon is due to impaired caspase‐3 activation during M‐phase downstream of mitochondrial outer membrane permeabilization (MOMP) in a fraction of mitochondria 7, 67 is entirely unclear, as these initial observations rather indicate a problem with G1/S transition 68, 69. Yet, these problems may originate in the previous M‐phase.

The fate of cells that actually escape mitotic arrest in the presence of microtubule poisons or targeted drugs by slippage is actually less well understood. Clearly, such cells are usually characterized by frequently failing cytokinesis and the presence of micronuclei that are a particular danger to genomic stability when the captured chromosomes or chromosome fragments re‐integrated after replication into the main genome. This can happen randomly in a shotgun‐like approach referred to as chromothripsis 70. In order to avoid this, the p53 network usually becomes activated which ultimately can trigger the death of such micronuclei‐baring cells or promote their senescence 71. Yet, the proficiency of this checkpoint seems to be variable and even cells carrying functional p53 may re‐enter the cell cycle, depending on the mitogenic load and their histone methylation status.

How p53 becomes activated in tetraploid G1 cells was subject of intense investigations. Recent evidence from our own group suggests that caspase‐2 is needed for this response and, by cleaving the E3 ligase, MDM2, usually degrading p53, at a conserved site, is stabilizing the transcription factor. Of note, this process is actually driven by the extra centrosome in such cells and requires assembly of the PIDDosome multiprotein complex that drives caspase‐2 activation 20, 72. In consequence, a unique p53 response is induced that is distinct from the one elicited by canonical DNA damage or extended mitotic arrest, as mentioned above 19. It can be assumed that such cells again build up an apoptotic activity that, in part, may depend on proteins modified in and carried over from the previous M‐phase 9, 11. What ultimately pushes the cell toward induction of apoptosis vs. cell cycle arrest and senescence under such conditions remains to be defined in full. Clearly, loss of p53, seen in many cancers, will ultimately preclude the activation of a set of cell death and cell cycle arrest regulators, facilitating the escape of such micronucleated tetraploid G1 cells, promoting the selection of more complex karyotypes culminating in aneuploidy tolerance, a major drive of tumor evolution (Fig 2).

## Molecular control of mitotic and post‐mitotic cell death

**Figure 3:**
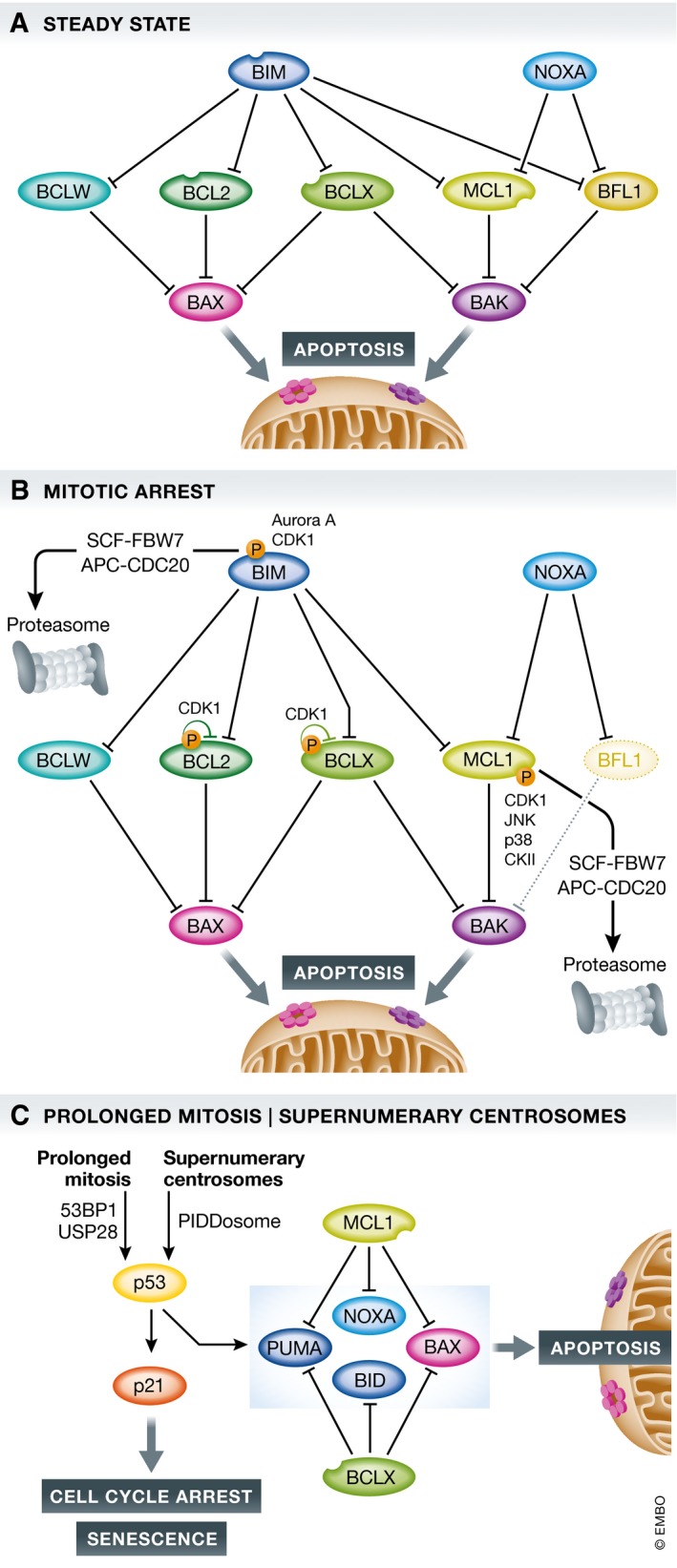
BCL2 family proteins implicated in the control of mitotic cell death or cell death after mitotic slippage (A) In healthy cells, a homeostatic equilibrium between cell death initiating BH3‐only proteins (blue), anti‐apoptotic BCL2 family proteins (green), and cell death executioners (pink/purple) is maintained. (B) Upon perturbation of this equilibrium, for example, by the action of anti‐mitotic drugs and prolonged arrest in mitosis, a series of events, including phosphorylation on and proteasomal degradation of pro‐survival BCL2 proteins, shifts the balance, favoring BAX/BAK1 activation. (C) Mitotic slippage or SAC adaptation can allow escape form mitotic cell death, yet newly initiated or carried over signaling cues impact on cell fate of such “post‐mitotic” cells. This can culminate in the induction of cell death, again potentially involving p53 plus a set of BCL2 family proteins that may have become active or changed in quantity in the preceding and prolonged M‐phase.

As pointed out above, the death of mitotically arrested cells is controlled predominantly by the BCL2 family that comprises a set of anti‐apoptotic molecules, including BCL2, BCLXL, MCL1, BCLW, and BFL1 (also known as A1), their antagonists of the “BH3‐only” subgroup, such as BIM, BID, PUMA, NOXA, BAD, and BMF, as well as the central regulators of MOMP, BAX, and BAK1 (reviewed in 73, 74). Their activation can be fine‐tuned by some BH3‐only proteins directly, most notably BIM and BID, that facilitate insertion into and dimerization of BAX or BAK1 in the mitochondrial outer membrane (MOM). BAX or BAK1 homodimers ultimately oligomerize into higher order structures that permeate the mitochondrial outer membrane (MOM) forming a toroidal pore 75, 76, allowing release of apoptogenic factors, such as cytochrome c, from the inner membrane space that control caspase activation for apoptotic cell death 74 (Fig 3A).

BCL2 family proteins have been studied early on in mitotic cell death and have been recognized as *bona fide* substrates of stress‐induced and mitotic kinases (see below). Yet, early findings were often highly contradictory, leaving behind a blurry picture related to the functional relevance of these modifications (reviewed in 77). Form the helicopter view, it seems all clear. Deletion of BAX and BAK1 blocks mitotic cell death (Fig 3B) and promotes mitotic slippage, albeit delayed cell division accompanied by chromosome segregation errors can also be observed 52. As apoptosis is abrogated in the absence of BAX and BAK1, either event should be followed by cell cycle arrest and senescence in the next tetraploid G1‐phase, at least in primary cells 78, 79. In the absence of p53, or a forced reduction in arrest proficiency by INK4a or p21 RNAi, the outcome becomes less clear and cells can continue to cycle and become aneuploidy 72, 78. Clearly, many such slipped tetraploid G1 cells do eventually die 10, 11.

In contrast to mitotic death, the molecular basis of this subsequent death in G1‐phase (Fig 3C) is poorly investigated and hard to dissect experimentally from death signals that may have been already initiated in the previous extended M‐phase, yet kill the cell only after mitotic exit. A possible means to address this is to compare the cell death machinery recruited by mitotic blockers with that of mitotic drivers used in combination with selective inhibitors of the BCL2 family. Such an analysis has been done and supports the idea that certain BCL2 proteins are critical for survival upon mitotic exit, in particular MCL1 and BCLX, at least in colorectal cancer cell lines 10, 11. Moreover, anti‐apoptotic BCL2 proteins were reported to be heavily modified and inhibited by post‐translational modifications (Table 1) or promoted for degradation by the proteasome, lowering the apoptotic threshold 77. Based on the minimalistic, *C. elegans*‐derived model of BCL2‐regulated apoptosis, supported in part also by mammalian cell line studies, inhibiting or removing all BCL2‐like pro‐survival proteins in a given cell can suffice to promote BAX activation and apoptosis 80. This, however, was raising the question whether BH3‐only proteins are actually needed to promote or fine‐tune apoptosis under conditions of extended mitotic arrest. An important note on the side here is that the trigger used to arrest cells in mitosis, for example, microtubule‐damaging agents or targeted anti‐mitotic drugs, does not seem to grossly affect the molecular mode of cell death execution upon extended mitotic arrest.

**Table 1:** Post‐translational modifications of BCL2‐family members

Protein	Residue modified	Kinase/PPase	Reported effects	Function	Reference No.
Anti‐apoptotic proteins					
BCL2	Undefined	Undefined	Phosphorylation noted during mitosis and taxol‐induced growth arrest; not involved in protein degradation	Unknown/not tested	101
	T69, S70, S87	JNK, ASK1	Observed during cell cycle progression to inactivate BCL2 at G2/M at S70; monitoring the fidelity of chromosome segregation	Pro‐apoptotic	102
	All	PP1 phosphatase	Observed during slippage; suggested to reactivate the anti‐apoptotic function of BCL2	Pro‐survival	107
	T56	CDK1/cyclin B	Phosphorylated during mitosis; found in nuclear structures in prophase; at later mitotic stages, P‐BCL2 localizes on mitotic chromosomes; relied on use of one particular antibody	Unknown/not tested	178
	Undefined	CDK1/cyclin B	During mitosis transient and incomplete phosphorylation of BCL2; sustained activity of CDK1 during mitotic arrest leads to increase in phosphorylation at multiple S/T sites; inactivation of anti‐apoptotic function; prevents association with BAX/BAK	Pro‐apoptotic	104 113 179
	S70	CDK1/cyclin B	Higher affinity to bind BAK1 and BIM in mitosis in cell‐free conditions; enhanced protection against apoptosis induced by chemotherapeutic drugs	Pro‐survival	93
	T56, S70	CDK1/cyclin B	Prometaphase arrest‐dependent; protein still localized in mitochondrial fraction irrespective of their phosphorylation; abrogates anti‐apoptotic function	Pro‐apoptotic	103
BCLX	S62	JNK	Taxol triggers phosphorylation during G2/M; increased apoptosis	Pro‐apoptotic	180
	S62	CDK1/cyclin B	Phosphorylation after vinblastine treatment; phosphorylation weakens interaction with BAX; phospho‐defective BCLX mutant retained ability to bind	Pro‐apoptotic	134
	S62	CDK1/cyclin B	During mitosis transient and incomplete phosphorylation of BCLX; sustained activity of CDK1 during mitotic arrest leads to increase in phosphorylation at multiple S/T sites; inactivation of anti‐apoptotic function; prevents association with BAX/BAK	Pro‐apoptotic	113
	S62, S49	PLK3	S62‐modified BCLX may interact with APC/C during mitotic checkpoint; S49 modified version seen during S‐ and G2‐phase but disappears in early mitosis—reappears during telophase; de‐stabilizes G2‐arrest and slows cytokinesis; accumulates at centrosomes in G2 after DNA damage; retains anti‐apoptotic effect	Unknown/not tested	114
	S62	PLK1/JNK2	Phosphorylated during normal cell cycle but accumulates in G2 after DNA damage; during that arrest phosphorylation at S62 promotes BCLX accumulation in nucleoli ‐> meets CDK1 and traps CDK1 to avoid timely entry into mitosis	Unknown/not tested	181
	S62	CDK1/cyclin B	Loss of BCLX function is the major driver of mitotic death; phosphorylation and degradation of MCL1 and phosphorylation of BCLX are both critical	Pro‐apoptotic	179
	Undefined	CDK1/cyclin B	CDK1/cyclin B is responsible for the strong phosphorylation of BCLX and BCL2 upon prometaphase arrest; only phosphorylation of BCLX activates intrinsic apoptosis	Pro‐apoptotic	108
	S62	Not defined	Observed during mitotic arrest; decreases affinity of BCLX for BAX ‐> impaired protection from mitotic cell death	Pro‐apoptotic	89
MCL1	S64	CDK1/2 or JNK	Observed during mitosis; enhances binding to BAK1, NOXA, and BIM; enhancing pro‐survival function; does not influence protein stability	Pro‐survival	182
	T92 + S64	CDK1/cyclin B	Seen during mitotic arrest; signal for APC/CDC20 to ubiquitinate MCL1 ‐> proteasomal degradation; S64 also phosphorylated in interphase but mutation of this site does not stabilize MCL1	Pro‐apoptotic	117
	S64, T92, S121, S159, T163	CDK1/cyclin B, p38, CKII, and JNK	T92 targeted by CDK1/cyclin B ‐> allows further phosphorylation by other kinases, leading to the recruitment of SCF/FBW7 for MCL1 degradation; phosphorylation on T159 and S163 reduces stability	Unknown/not tested	118
	T92	CDK1/cyclin B	Seen during mitosis, induces proteasomal degradation; release of bound BAK for apoptosis	Pro‐apoptotic	121
	S64, T68, T70, T92, S121, T156, S159, S162, T163	For novel sites: GSK3, CDK1, AMPK	CDK1‐mediated phosphorylation at T92 is dispensable for mitotic arrest‐induced MCL1 phosphorylation and degradation; mutation of five putative CDK1 sites to alanine cannot prevent degradation of MCL1	Pro‐apoptotic	122
Pro‐apoptotic proteins					
BAX	S184	AKT/PKB	Inhibits apoptosis, sequestration of BAX in the cytoplasm, heterodimerizes with MCL1, BCLX, or BFL1	Pro‐survival	136
BAK	T108	BMX	Inhibition of autoactivation	Pro‐survival	137
BIM	S69	ERK1/2	ERK‐mediated ‐> induces ubiquitination and SCF/βTrCP1‐dependent degradation during mitotic arrest	Pro‐survival	139
	S55, S65, S73, S100, T112, S114		Mutating all sites abrogates phosphorylation of BIM during mitosis; CDK1 but not ERK1/2 or ERK5 phosphorylates BIMEL during mitosis	Unknown/not tested	143
	S93, S94, S98	PP2A phosphatase	Reverses phosphorylation by Aurora A, de‐phosphorylation stabilizes BIMEL after mitotic exit	Pro‐apoptotic	140
BID	S66	CDK1/cyclin B	Phosphorylated as cells enter mitosis, phosphorylation lost during transition from meta‐ to anaphase; sensitizes mitochondria for MOMP during mitotic arrest	Pro‐apoptotic	85
BAD	S128	Undefined	Enhances the apoptotic activity during mitosis; dominant negative mutant fails to prevent taxol‐induced apoptosis	Pro‐apoptotic	151

A number of more recent studies came to similar conclusions regarding the initiation phase of mitotic cell death, that is, a subgroup of BH3‐only proteins is involved in activating BAX and BAK1 in mitotically arrested cells. The Taylor group conducted a genome‐wide RNAi screen defining candidates that promote cell death in mitosis 11. Next to a group of genes controlling mitotic entry, including cyclin B, CDK1, and EMI1, they also noted a selection against MYC. In follow‐up analyses, they found evidence that the ability of MYC to increase levels of certain BH3‐only proteins, BIM, BID, and NOXA, was critical to determine the fate of mitotically arrested cells and that MYC‐driven repression of BCLX favored apoptosis in mitosis (Fig 3B). Consistently, chemical inhibition of BCLX sensitized cells with low MYC levels toward mitotic as well as post‐mitotic cell death in different epithelial cancer cells 11. The sensitizing effect of BCLX inhibition for mitotic cell death has been noted before by the Mitchison group using RNAi and a broad‐spectrum BCL2 inhibitor, ABT‐263, that also blocks BCLX and BCLW 81. RNAi against BCL2 or BCLW had minor sensitizing effects in some cells (e.g., U2OS), but showed no effect in others (e.g., A549). BCLW is an understudied BCL2 pro‐survival homologue that is found frequently expressed in epithelial cancer cells and that may act redundantly with BCLX. Silencing by RNAi was shown to accelerate mitotic cell death also in HeLa cells 82, a phenomenon also seen by Shi and colleagues 81.

Of note, in another RNAi‐based screening approach for regulators of mitotic checkpoint adaptation and slippage, the Yu laboratory concluded a critical role for NOXA in cell death, but the authors did not find a significant contribution of BIM 50, that has been implicated in this process before by others 83, 84. We conducted an RNAi‐based mini‐screen in HeLa cells to address the question whether and which BH3‐only proteins may be involved in mitotic cell death and identified BIM and NOXA as potential modulators of BAX and BAK1 activation in this setting 52. Together with our analysis of post‐translational modifications on BCL2, BCLX, and MCL1, as well as MCL1 protein turnover, we propose here an adopted M‐death model to explain the role of the BCL2 family network in deleting cells arrested for extended periods of time in mitosis (Fig 3B). Our model finds clear support by the findings of the Taylor, Mitchison, and Yu laboratories (discussed in detail below). We propose that CDK1‐mediated phosphorylation of BCL2 and BCLX potentially serves to lower the threshold for apoptosis induction to a certain degree by changing relative binding affinities while degradation of MCL1 acts as the actual molecular timer. MCL1 degradation is driven by NOXA (see below) that liberates BIM from sequestration by MCL1 to take out BCLX (and potentially BCLW; not tested by us), promoting death in mitosis. The contribution of BID to this process may be cell type‐dependent 85 or become critical only upon mitotic exit 86, as may be the contribution of other BH3‐only proteins not scoring in any of the RNAi screens conducted but able to act in a redundant manner with BIM, most notably PUMA 87 or BMF 88. Finally, similar to BCLX, MCL1 levels appear to be particularly important for cells which survive mitotic arrest by slippage as its depletion significantly increases cell death in such cells 54, 86. Similarly, cells with low‐level expression of MCL1, such as DLD1 colon cancer cells, or cells where MCL1 levels were reduced by RNAi are highly sensitive to BCLX inhibitors when combined with mitotic drivers, such as the Aurora B inhibitor ZM447439 10, 86.

What remains obscure is that most studies actually failed to detect a significant contribution of BCL2 for the survival of M‐arrested cells, as neither RNAi‐mediated ablation nor chemical inhibition using ABT‐199 sensitized epithelial cancer cells efficiently to death in mitosis 81, 82, 89 while overexpression of BCL2 clearly protects 9, 52, 90. One possible explanation may be that BCL2 binds poorly to BAK1, and this may be the preferred executer of mitotic cell death, as it will be liberated and activated upon MCL1 degradation, restrained then only via BCLX in this setting. This may also explain the sensitizing effects of WEHI‐539, selectively targeting BCLX, toward anti‐mitotic drugs. On the other hand, ABT‐199 is most effective in leukemia which is more often BCL2‐dependent, such as B‐cell‐derived chronic lymphocytic leukemia (CLL) 91 and some types of acute myeloid leukemias 92. Sensitizing effects of RNAi‐mediated ablation or ABT‐199 treatment have been reported in K‐562 and Jurkat leukemia cell lines treated with vinca alkaloids 93. Hence, the relative dependence on BCLX vs. BCL2 for survival in mitotic arrest seems to be cell type‐dependent and co‐defined by the set of BH3‐only antagonists expressed or activated under these conditions.

Alternative modes of cell death are well established. So far, the evidence that death receptors and its ligands, such as FAS/FASL, TNFR1/TNF, or TRAILR/TRAIL, all members of tumor necrosis factor receptor/ligand superfamily that depend on caspase‐8 to mediate apoptosis or promote cell survival do play a role here is limited. However, non‐apoptotic functions of caspase‐8, its antagonist, c‐FLIP, and their adapter, FADD, have been reported to regulate proliferation rates upon mitogenic stimulation of splenocytes and in hepatocytes 94, 95. If anything, these receptor/ligand pairs may be engaged to induce or contribute to inflammatory signaling under conditions of mitotic arrest or upon slippage in tetraploid G1 cells that may become senescent 71, 78, 79. Similarly, necroptosis, a form of cell death engaged downstream of these receptors when caspase‐8 is blocked 96, has not been linked to mitotic or post‐mitotic cell death so far. Caspase‐2, on the other hand, has been implicated in mitotic catastrophe a long time ago 97, as well as in the execution of cell death induced by anti‐mitotic drugs 98. Yet, while it does not significantly contribute to mitotic cell death 72, 99, it eventually may be involved in the initiation of post‐mitotic cell death via mechanisms that remain to be defined in full. This response might involve the BH3‐only protein BID 13, 85 that can be processed and activated by caspase‐2 100.

## Post‐translational modifications of pro‐survival BCL2 family proteins and their relevance for mitotic cell death and cell death after slippage

**Figure 4:**
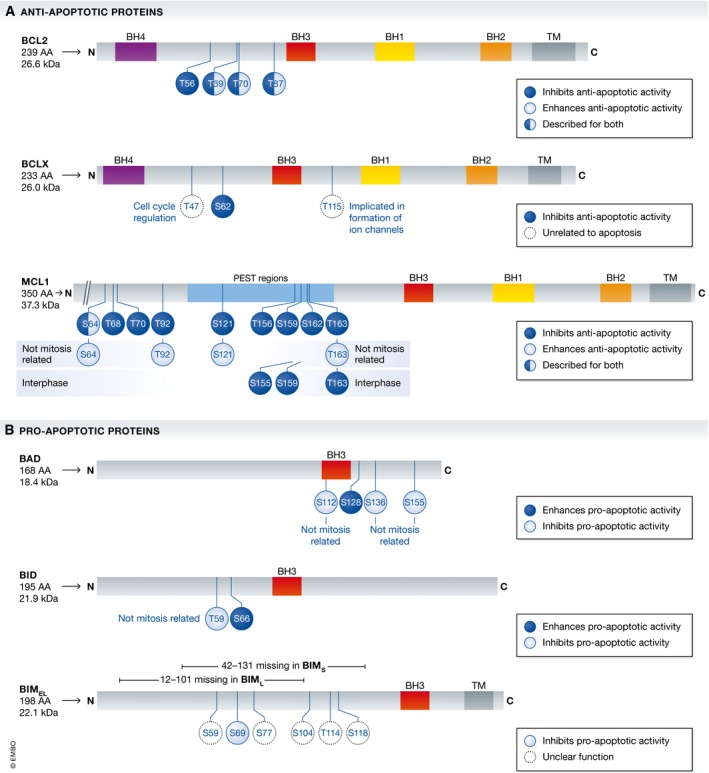
BCL2 family proteins targeted by phosphorylation in mitosis The BCL2 protein family controls cell death upon extended mitotic arrest. Multiple phosphorylation events of pro‐ and anti‐apoptotic family members have been found in mitosis. Not all of them have been functionally characterized, but generally, it is believed that phosphorylation on pro‐survival proteins reduces their function, while the same type of modification promotes the death function of pro‐apoptotic BH3‐only proteins. A detailed list of reported phosphorylation events in and out of mitosis, their potential impact on function, the kinases and phosphatases involved, and the related reference can be extracted from Table 1.

The founding member of the family, BCL2, was described early on to be phosphorylated during mitotic arrest 101. Multiple phosphorylation sites were identified, including T69, S70 and S87 102, T56 103, and possibly also T74 93, all located in an unstructured loop of the protein between alpha 1 (BH4 domain) and alpha 2 (BH3 domain). Phos‐tag gel analysis actually suggests that these phosphorylation events are quantitative as even exogenously overexpressed BCL2 gets fully phosphorylated in M‐arrested HeLa cells 52. The exact role of these phosphorylation events remains controversial however (Fig 4A, Table 1). Through the use of point mutations of the various residues, generating either phospho‐defective alanine mutations or phospho‐mimetic glutamate mutations, some studies have concluded that phosphorylation of BCL2 is inhibiting its pro‐survival function 52, 102, 104, while others reported on enhanced anti‐apoptotic function 68, 105. One report actually suggests that a conformational change in the normally unstructured loop region rather than the inserted charge affects BCL2 structure and behavior. Mutagenesis at position S70, usually targeted by CDK1 for phosphorylation, increased the binding affinity of BCL2 for BAK1 and BIM in surface plasmon resonance analyses and increased cell death protection in leukemia cells regardless of whether a negatively charged or aliphatic amino acid was introduced 93.

Another possible effect investigated was BCL2 protein stability after its phosphorylation when MCF7 cells were challenged with paclitaxel 106. Non‐phosphorylated BCL2 showed reduced half‐live, though we and others did not note degradation of BCL2 during mitotic arrest 52, 90. The kinase most frequently implicated in modifying BCL2 is CDK1 as it was able to phosphorylate BCL2 in cells 93 and the CDK inhibitor roscovitine was able to prevent this 103, 105. After escaping mitotic arrest by slippage BCL2 is rapidly de‐phosphorylated by PP1 phosphatase 107. Even though BCL2 overexpression can protect cancer cells effectively from mitotic death 52, 90, combinatorial treatment of anti‐mitotic drugs with inhibitors selectively targeting individual BCL2 family proteins suggests that, at least in epithelial cancer cells, BCLX is more critical than BCL2 for survival during and possibly after slipping from extended mitotic arrest 81, 89, 93, 108. In conclusion, the physiological relevance of BCL2 phosphorylation for cell death initiation in this context remains uncertain. It is tempting to speculate that some of these phosphorylation events on T69, S70, and S87, reported to abrogate also interaction with the autophagy regulator Beclin1 in response to starvation 109, 110, may serve to facilitate the activation of cell death by alternative means. Studies by Domenech and colleagues actually suggest that this initially protective and energy‐restoring response can contribute to cell death during extended mitotic arrest 53. Regardless of the ultimate truth, it seems fair to say that phosphorylation of BCL2 in mitosis does not substantially modulate its core survival function or interaction pattern with other BCL2 family proteins, but may act to fine‐tune the apoptotic threshold by controlling affinities of established interaction partners.

Like BCL2, BCLX is readily detectable as being phosphorylated in a highly quantitative manner during mitotic arrest and similar to BCL2, remains highly stable 111. It was shown that the higher molecular weight complexes containing BCLX found in normal cells in cross‐linking experiments are readily lost after treatment with microtubule‐targeting agents 112. The only mitotic phosphorylation site characterized in BCLX so far, on S62, again contained in the unstructured loop of the protein between helices alpha 1 and alpha 2, is also targeted by CDK1/cyclin B 113. The effect of this phosphorylation has been shown to inhibit the anti‐apoptotic potential of BCLX 89, 113. Numerous alternative phospho‐sites in BCLX have been described, such as phosphorylation on Ser49, targeted by polo‐like kinase (PLK)3, but this modification is not detectable prior telophase 114. Yet, these phospho‐sites appear to be critical for cell cycle fidelity, as overexpression of site mutants negatively affects chromosomal stability in diploid fibroblasts 115. The consequences of BCLX phosphorylation on complex formation with either BAX/BAK1 or BH3‐only proteins, however, are poorly understood (Fig 4A, Table 1).

Without doubt, MCL1 seems to be the most crucial survival factor within the BCL2 family, as suggested by multiple studies investigating consequences of its loss by genetic means (reviewed in 116). Again, MCL1 has been shown to be subjected to intense phosphorylation events during mitotic arrest (Fig 4A, Table 1), but in stark difference to BCL2 and BCLX, this phosphorylation has been reported as the prerequisite for the degradation of MCL1 in mitosis. The first report on this topic demonstrated phosphorylation on S64 and T92 by CDK1/cyclin B, with mainly T92 leading to the degradation of MCL1 in the proteasome after ubiquitination by the APC/C‐CDC20 E3‐ligase complex 117. Later, researchers at Genentech defined the mitotic phosphorylation events on S64, S121, S159, and T163, with only the latter two influencing the stability of MCL1 118. The publication identified JNK1, p38, CKII, and CDK1 as kinases involved in phosphorylation and the SCF‐FBW7 complex as the executing E3 ubiquitin ligase degrading MCL1 in mitosis and as a key factor determining sensitivity to anti‐mitotic drugs in cancer. At the same time, another group found that two of the sites, S159 and T163, can be phosphorylated by GSK3, which targets MCL1 for SCF‐FBW7‐dependent degradation 119. However, this was described in asynchronous, cycling cells without any mitotic inhibitors. Consistent with these reports, tumors that have lost FBW7 function were shown to be more resistant to anti‐mitotic agents and showed poorer prognosis 118, 119.

JNK‐driven phosphorylation 120 and CDK1‐driven phosphorylation 121 were also found by other groups to be important for the de‐stabilization of MCL1 in mitotically arrested cells. The role of the reported phosphorylation sites, however, has been put into questions lately. A recent study interrogated all previously defined mitotic phospho‐sites on MCL1 in mitosis, including three newly defined ones coming to a surprising conclusion: The pan phospho‐defective alanine mutant version of MCL1, carrying 9 alanine substitutions, was degraded as rapidly as the wild‐type version of the protein during mitotic arrest 122. Additionally, the E3 ubiquitin ligases described to degrade MCL1 in mitosis were equally challenged in a recent study 54 as the authors were unable to stabilize MCL1 with either the APC/C‐CDC20 inhibitors apcin and proTAME nor knockdown of SCF‐FBW7. Together, these data suggest that MCL1 turnover is executed by different E3 ligases in a highly redundant manner and that phosphorylation events have little impact on MCL1 protein stability in mitosis in most cells. Along these lines, it is worth mentioning that an “all KR” mutant of MCL1 is degraded as effectively as wild‐type MCL1 in cells after cycloheximide treatment 123, suggesting either a role for N‐terminal ubiquitination, acting as a backup, or alternative routes of protein degradation, for example, directly in the 20S‐proteasome or, potentially, in lysosomes.

Independently of E3 ligases and phosphorylation events, it was shown that the BH3‐only protein NOXA promotes the degradation of MCL1 in mitosis and thereby defines the sensitivity of epithelial cancer cells to anti‐mitotic drugs 52. Similarly, the Yu laboratory identified NOXA in an RNAi‐based screen for factors controlling SAC proficiency, linking it also to MCL1 inhibition and as a target of the pro‐survival role of p31comet, controlling SAC duration 50. It is worth mentioning that NOXA levels accumulate most in G2 and drop again in M and upon mitotic exit in HeLa cells 52. Yet, in M‐arrested cells, NOXA gets co‐degraded with MCL1 and its ablation by RNAi leads to increased MCL1 stability and prolonged survival 50, 52. Of note, also a KR mutant version of NOXA appears to be degraded with similar kinetics than wild‐type protein 124. As NOXA and MCL1 are only degraded effectively when complexed with each other 125, all these studies interrogating the individual proteins may have failed because none of them investigated the half‐life of KR versions of both proteins complexed together in one cell, or because of NOXA as an intrinsically unstructured protein, dragging the complex directly into the 20S proteasome.

Surprisingly, under conditions of NOXA RNAi, BIM levels increase and it is tempting to speculate that this is due to sequestration by MCL1 and/or BCLX. Accordingly, simultaneous knockdown of BIM and NOXA prolongs survival of mitotically arrested cells, while inhibition of BCLX promotes death in mitosis 10, 11, 81. Clearly, NOXA controls MCL1 stability, and consistent with a timer function of MCL1 in mitosis, miRNAs that affect MCL1 protein levels, such as miR29b and miR101, were shown to modulate sensitivity to microtubule‐targeting agents 126, 127. Unexpectedly, however, simultaneous depletion of NOXA and BIM did not only affect time to death upon mitotic arrest but also increased the mitotic duration until slippage 52. This finding implicates a certain cross talk between the two competing networks.

BCLW, mainly known for its essential role in spermatogenesis 116, has been neglected in most studies investigating mitotic cell death. However, BCLW can play a role in setting the apoptotic threshold in HeLa or U2OS cells arrested in mitosis as siRNA against or knockout of BCLW shortens the duration until mitotic death 81, 82. No post‐translational modifications have been reported on BCLW in mitosis so far. It was shown that BCLW is a direct target of miR107 and that this miRNA is downregulated in a taxol‐resistant A549 derivative when compared to the parental cell line 128. Yet, clearly, this effect of miRNA repression may involve de‐repression of alternative targets, next to BCLW. Lastly, similar to BCLW, BFL1/A1 is still understudied, when compared to the other pro‐survival BCL2 family members mentioned so far. While protective roles from multiple apoptotic stimuli activating mitochondrial cell death were reported when BFL1 is overexpressed, nothing is known about its role in mitotic cell death, nor have PTMs been analyzed in that context. Clearly, BFL1 has a short half‐life affected by ubiquitination 129, 130. However, it might be an attractive target in some types of blood cancer, such as diffuse large B‐cell lymphoma (DLBCL) or myeloid malignancies as well as melanoma skin cancer, as these tumors appear to frequently use aberrant expression of BFL1 to secure survival 131. Notably, it was shown that BFL1 knockdown, in contrast to BCL2 inhibition, sensitizes a B lymphoblastoid IM9 tumor cell line, expressing low levels of MCL1, to vincristine 132. Furthermore, all‐trans retinoic acid (ATRA) protects NB4 acute promyelocytic leukemia cells from taxol‐mediated cell death by upregulating BFL1 133 suggesting a role in mitotic cell death in some cell types.

## Post‐translational modifications of pro‐apoptotic BCL2 family proteins and their relevance for mitotic cell death and cell death after slippage

BAX and BAK1 are redundant executers of MOMP, and hence, treatment of cells with mitotic inhibitors eventually culminates in their activation and oligomerization prior cell death 112, 134. Accordingly, BAX and BAK1 deficiency or depletion results in a significant survival benefit during mitotic arrest and promotes a strong slippage phenotype. In some cellular systems, BAK1 seems to be playing a dominant role 52, 134, 135, consistent with its preferential interaction with MCL1, when compared to BAX 74. Little information is out there if BAX or BAK1 are undergoing PTMs in mitosis and if such modifications modulate their activity during mitotic arrest. Clearly, survival kinases such as AKT can phosphorylate BAX on S184 to restrain its pro‐death function 136 and phosphorylation on T108 of BAK1, elicited, for example, by the tyrosine kinase BMX, antagonized by PTPN5 phosphatase, has been shown to negatively regulate BAK1 oligomerization activity 137, 138. Whether any of these or other residues are modified in mitosis is unknown. It is also unknown whether BAX/BAK1 DKO cells that escape mitotic arrest by slippage eventually die by non‐apoptotic cell death, become genomically instable or simply undergo senescence.

While BAX/BAK1 is essential for mitochondrial apoptosis despite being functionally redundant (Fig 3A), the large group of pro‐apoptotic BH3‐only proteins are usually highly redundant and none of them is essential for apoptosis, nor is any combination tested genetically so far (reviewed in 73). BIM is the most thoroughly investigated BH3‐only protein, including during mitotic arrest (Fig 4B, Table 1). What remains undisputed is that BIM is heavily phosphorylated during mitotic arrest. It was shown that the residues S69 139 as well as S94 and S98 are phosphorylated by Aurora A kinase with the latter two residues leading to ubiquitination and SCF‐βTrCP1‐dependent degradation during mitotic arrest 140. Counterbalancing this is the action of the phosphatase PP2A since its inhibitor, okadaic acid, or siRNA against this phosphatase drastically increases the phosphorylation and de‐stabilizes BIM 140. The Aurora A‐dependent degradation of BIM by SCF‐βTrCP1 is somewhat at odds with a report documenting direct binding of BIM to the APC/CDC20 complex in late mitosis, targeting it for proteasomal degradation 141. Again, chemical inhibition of the APC or CDC20 RNAi leads to an accumulation of BIM that was identified to carry two destruction (D)‐BOX motifs in position 72–87 142. The binding between BIM and APC/CDC20 was shown to be counteracted by the phosphorylation of BIM on S83, most likely mediated by PKA. This notion is at odds with the observation that the mutation of six different residues (S55, S65, S73, S100, T112, and S114), considered to be targets of CDK1/cyclin B1 in mitosis, abrogated the mobility shift of mitotic BIM protein seen in SDS–PAGE 143. Moreover, while there is supportive evidence for a cell death executing role of BIM in cells challenged with mitotic inhibitors 52, 84, 144, some studies failed to recapitulate these findings 135, 139. These differences may often be due to alternative experimental design or knockdown efficiency in RNAi experiments. Along that lines, the transcription factor FOXO3—controlling *BIM* transcription—was shown to mediate sensitivity to long‐term taxol treatment in MCF‐7 breast cancer cells 144. As transcription supposedly stops in mitosis, it remains plausible that BIM levels in post‐mitotic G1 cells that may have escaped mitotic arrest are controlled by FOXO3 that may bookmark the gene locus already in M‐phase and thereby control overall drug sensitivity. Furthermore, ERK—while probably not responsible for the mitotic phosphorylation of BIM 143—can confer resistance to low doses of microtubule‐targeting agents. ERK inhibition together with low dosage of MTAs caused dramatic BIM‐dependent mitotic death 145. This, however, may be explained by increased baseline levels of BIM, due to ERK inhibition that may sensitize HT1080 cells to taxol treatment in mitosis and potentially other cell death triggers. Together, these studies document that BIM is setting a threshold for cell death in mitosis and that this threshold is fine‐tuned by BIM phosphorylation that can happen before or upon mitotic arrest (Fig 4B, Table 1).

As discussed in part already above, NOXA is a critical player in the execution of mitotic cell death as its depletion by siRNA is able to give a significant survival benefit upon extended mitotic arrest 50 and profoundly impairs the mitotic degradation of MCL1 52. This potentially leads to reduced BIM activity, as it accumulates in NOXA RNAi cells. As BIM can interact with comparable affinity with all BCL2 pro‐survival homologues, it is most likely then bound by excess MCL1, free BCL2, BCLX, or BCLW, depending on the cell type‐dependent makeup. How NOXA protein levels are regulated during cell cycle progression is unclear, as is the question whether it gets post‐translationally modified before or during mitotic entry. CDK5‐mediated phosphorylation of NOXA has been reported to control its localization and the cellular response to glucose deprivation 146. Of note, inhibiting glycolysis using inhibitors of PFKBP3 is speeding up mitotic cell death in combination with taxol 53. Clearly, NOXA levels are highest in G2‐phase, before getting co‐degraded with MCL1 in mitosis. Together with BIM, NOXA promotes caspase activation and double deficiency or combined targeting by RNAi increases the time to death in mitotically arrested cells 11, 52. Yet, ultimately, these cells do die in mitosis, as the rate of slippage was not significantly affected, suggesting additional redundancies or compensatory activation of other BH3‐only proteins. Whether and how de‐ubiquitinating enzymes reported to regulate stability of BIM 147, NOXA 148, or MCL1 149 affect mitotic cell fate remains to be formally tested.

Genetic analysis in mice has demonstrated beyond doubt that BH3‐only proteins usually act in a redundant manner 73, and hence, it is not surprising that other members of this subgroup may contribute in one or the other cell type or experimental setting. Of note, the BH3‐only protein BID, usually considered to link extrinsic to intrinsic apoptosis pathways upon caspase‐8‐mediated proteolysis 150, appears to undergo an activating phosphorylation event during mitotic arrest at S66 by a yet to be identified kinase (Fig 4B, Table 1) in the absence of caspase‐mediated processing, promoting its mitochondrial localization 85. Furthermore, depletion of BID could protect cells from the toxic effects of mitotic inhibitors 85 but this effect was not seen by others 52 and hence may be an either cell type‐specific effect or affecting cell death after slippage 13, 86. Finally, the BH3‐only protein BAD can become phosphorylated during mitotic arrest at S128 in a CDK1‐ and JNK‐dependent manner (Fig 4B, Table 1), but the relevance for mitotic cell death remains unclear 105, 151. Usually, phosphorylation of BAD at other residues, such as S112, S136, or S155, is inhibitory and mediated upon growth factor or cytokine addition via the kinases PKA or PKB/AKT 152. S128 phosphorylation, however, appears to interfere with these inhibitory phosphorylation events and reduces binding of BAD to 14‐3‐3 proteins that retain them in the cytoplasm 151. Little is known about the remaining members of the BH3‐only subgroup. One study suggests that the BH3‐only protein BMF may promote BIM‐mediated breast cancer cell death in response to taxol, yet it is unclear whether this is happening during mitotic arrest or upon slippage 90. As BMF has a more restricted expression pattern as BIM, its contribution may be cell type‐dependent 88. No meaningful information is currently available for the ER‐stress engaged BH3‐only protein BIK/NBK 153 in mitotic cell death and HRK, preferentially found expressed in post‐mitotic neurons 154.

While the role of BH3‐only proteins in mitotic cell death has been investigated to a reasonable degree, their role in cell death upon slippage is less clear. As indicated above, BID might be relevant for post‐mitotic death upon SAC override using the MPS1 kinase inhibitor reversine in p53‐deficient cells as a substrate for caspase‐2 13. Yet, BID cleavage can also be observed in colon cancer cell lines proficient for p53 where slippage has been induced by Aurora B kinase inhibition, rendering them highly dependent on BCLX for survival. These cells are further characterized by an accumulation of NOXA, PUMA, and/or BIK. Knockdown of NOXA by RNAi clearly protected these cells from cell death upon slippage in the presence a BCLX inhibitor, as did knockdown of BAX, while RNAi against the other BH3‐only proteins induced was not protective 86.

## Linking mitotic errors to inflammation and immunity

DNA damage can arise in response to chromosome segregation errors in various ways. For example, physical constriction of lagging chromosomes in the cleavage furrow during cytokinesis elicits a canonical DNA damage response in daughter cells inheriting damaged chromosomes or chromosome fragments 155. Alternatively, DNA damage arises within micronuclei after whole‐chromosome missegregation during asynchronous replication and subsequent chromosome fragmentation 70. It is well‐appreciated that DNA damage can cause activation of NF‐κB signaling triggering a subsequent inflammatory response leading to cytokine and chemokine release 156. These cytokines can alert the immune system that aids the efficacy of anti‐cancer therapy. In contrast to pathogen‐driven inflammatory cytokine‐mediated activation of NF‐κB, this sterile inflammatory response appears to occur with delayed and often bimodal kinetics but is mechanistically poorly understood. Early studies suggest involvement of RIPK1‐regulated signal transduction as well as a need for ATM‐kinase‐mediated phosphorylation of NEMO/IKKγ and PARP1 activity 157, 158, 159. Moreover, PIDD1, discussed above in the context of p53 activation, has been suggested to act as a missing link connecting DNA damage with RIPK1 and NEMO‐driven NF‐κB activation 160, 161. Yet, how damaged DNA is actually sensed to trigger this inflammatory pathway remained largely unclear.

**Figure 5:**
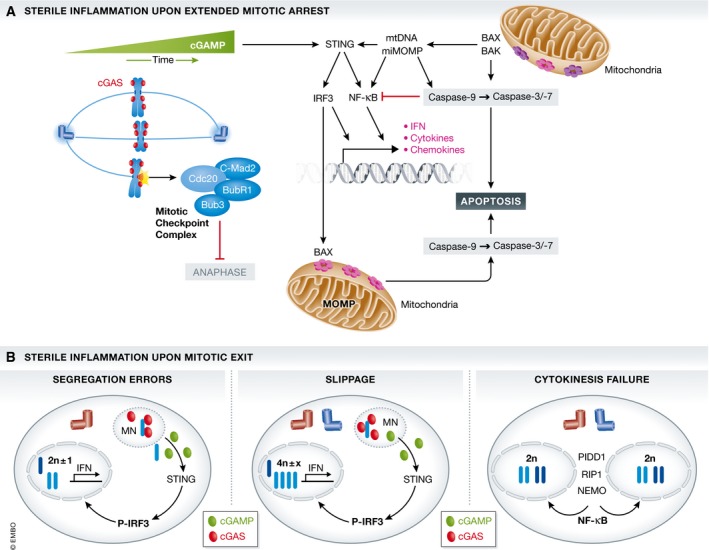
Linking mitotic arrest and slippage to inflammation and immunity (A) The dsDNA immune sensor cGAS, found associated with chromatin in mitosis, can synthesize a second messenger cyclic AMP/GMP from ATP and GTP that activates an ER‐resident signaling molecule, stimulator of interferon genes (STING). STING can trigger the activation of transcription factors, including the interferon response factor (IRF)3 and NF‐κB, leading to the production type I interferons, IFN, as well as a set of inflammatory cytokines and chemokines to alert the immune system and neighboring cells. A non‐transcriptional cell death activating function of IRF3 at mitochondria may contribute to mitotic cell death directly. Moreover, mitochondrial outer membrane permeabilization in a minority of mitochondria (miMOMP), frequently seen during mitotic arrest, could lead to NF‐κB‐driven inflammation when caspase activation is impaired while released mitochondrial (mt)DNA could lead to STING‐dependent IFN production. (B) Errors in mitosis that lead to micronucleation in the next G1‐phase, such as chromosome missegregation or slippage, alert the immune system via recruiting the cGAS/STING signaling pathway, described above. cGAS enters micronuclei upon lamin breakdown, binds to nucleosomal chromatin, and produces the second messenger cGAMP for STING activation and IFN signaling. Cytokinesis failure that does not lead to micronucleation, yet tetraploidy, may engage pro‐inflammatory signaling via the activation of the so‐called NEMO–PIDDosome complex, leading to NF‐κB activation.

Nucleic acids, including double‐stranded (ds) DNA of foreign or self‐origin, have been recognized as pathogen‐ or danger‐associated molecular patterns (PAMP/DAMP), respectively, and different sensory systems have developed during evolution to alert the immune system in their presence. Toll‐like receptor (TLR)9 162 as well as cytoplasmic cGAS 163 can respond to lysosomal or cytoplasmic dsDNA, respectively, inducing NF‐κB and interferon signaling. Cytoplasmic dsDNA elicits a subsequent interferon response by engaging ER‐resident STING via the cGAS‐synthesized second messenger, the cyclic dinucleotide cGAMP, to promote TBK1‐dependent phosphorylation and activation of the transcription factor IRF3 163. Keeping this in mind, one may actually wonder why the cGAS/STING pathway sensing cytosolic DNA is not activated automatically during mitotic cell division when DNA is present in large amounts and unshielded in the cytoplasm. Even more intriguingly, cGAS can actually bind to nucleosome‐bound DNA yet appears less active in this context [preprint: 164]. While this phenomenon may be due to inhibitory signals preventing full activation of cGAS that remain to be discovered in normal mitosis, it has been recognized that cGAS indeed becomes activated in response to DNA‐damaging cues, including irradiation, but also after nocodazole treatment, usually arresting cells in mitosis 18, 165. Remarkably, the activation of this pathway required mitotic traverse and was linked to the formation of micronuclei, as a consequence of these insults (Fig 5A). Moreover, even sterile means to increase micronuclei formation, such as genetic alterations in RNase H2, associated with autoimmunity in humans, favoring formation of chromosome bridges, promoted a cGAS/STING‐driven inflammatory response 165, 166. This was nicely corroborated by single‐cell RNAseq analysis of cells with or without micronuclei 165. Importantly, the nuclear envelope forming around micronuclei is notoriously unstable and prone to disassemble due to lamin breakdown, leading to early recruitment of cGAS. This interaction allows synthesis of the second messenger cGAMP and the induction of inflammatory genes in a STING‐dependent manner 163. It is intriguing to note that STING is an ER‐resident protein and that breakdown of nuclear membranes surrounding micronuclei allows chromatin to become invaded by underlying ER membranes 167, bringing all components needed for signaling into close proximity. Together, these recent studies show how genomic instability is linked to the immune system and explain why cancer cells that are often prone to micronucleation, for example, due to p53 loss, select against expression of components of this signaling axis, that is, in order to avoid sterile inflammation and immune cell activation (Fig 5A).

What does all this have to do with mitotic cell death? Not much, possibly post‐mitotic death at best, or post‐mitotic induction of senescence and the associated senescence‐associated secretory phenotype (SASP) 168, 169, 170. The latter may ultimately lead to the activation of death receptor signaling. Yet, one recent report on bioRxiv actually provides evidence that upon extended mitotic arrest, cGAS can be detected along mitotic chromosomes and that blocking cGAS/STING signaling delays mitotic death upon taxol treatment when slippage is blocked by APC/C inhibition [preprint: 164]. Intriguingly, cell death appears to involve a non‐transcriptional activity of IRF3 that has previously been reported in the context of infection with dsRNA viruses, that is, to be able to directly activate BAX via protein–protein interaction at the mitochondrial outer membrane 171. Of note, IRF3 contains a structural motif that closely resembles a BH3 domain and that may facilitate BAX oligomerization, in a way similar to certain effector BH3‐only proteins 172. Clearly, pro‐death effects of cGAS/STING agonists have been reported meanwhile in many different settings, including T 173‐ and B‐cell leukemia 174. Yet, the molecular mode of cell death remains to be fully defined (see Box 1).

## Implications for cancer therapy

One is tempted to speculate that drugs targeting mitotic kinases, microtubule motors, or the APC/C may have failed in the clinic so far, because they kill cells in mitosis too effectively. Classical microtubule‐targeting agents, on the other hand, are only moderate killers promoting slippage and micronuclei formation around lagging chromosomes, thereby leading to treatment‐associated inflammation in the next G1‐phase. It remains to be noted once more that clinically achieved doses of microtubule‐targeting agents are ineffective in killing cancer cells in mitosis *in vitro*, but promote subsequent multipolar cell divisions, chromosome missegregation, and micronuclei formation 15. *In vivo*, this may amplify inflammatory response, triggering strong anti‐tumor immune responses affecting the efficacy of anti‐mitotic drugs, but also the effectiveness of immunotherapy, as shown most recently 18. While early on during treatment, CIN and subsequent micronucleation may be beneficial for treatment responses because of its pro‐inflammatory nature (Fig 5B), aneuploid karyotypes may be selected that eventually allow tumor cell survival and immune escape. Again, such a phenomenon has already been documented in melanoma patients subjected to immunotherapy. In a retrospective analysis of clinical trials, Davoli and colleagues noted an inverse correlation between the degree of aneuploidy, tumor immune cell infiltration, and treatment response of melanoma patients 175. Consistently, hyperploid cancer cells, prone to become aneuploidy more easily, show an ER‐stress response due to proteotoxic stress and are eventually cleared by the immune system 176.

While it has become clear that mitotic cell death is controlled by the BCL2 family and that blocking individual BCL2 pro‐survival factors using BH3 mimetics can actually increase the efficacy of anti‐mitotic drugs, it remains to be seen whether clinical benefit can be improved. In light of the above, one could also envision that any strategy that increases mitotic cell death will eventually fail to increase therapy success, as only apoptotic death that is already poorly immunogenic per se will be enhanced, while curtailing secondary inflammatory events. As such, one could also postulate that drugs that promote cell death in the next G1‐phase in cells that escape mitotic arrest, either as micronucleated tetraploids or as aneuploidy diploids, might be clinically more successful. Therefore, mitotic drivers, such as MPS1 or Aurora B inhibitors that foster segregation errors, and, indirectly, subsequent micronuclei‐driven inflammation seem interesting alternatives to be combined with BH3 mimetics and immune checkpoint blockade for more effective anti‐cancer therapy. Alternatively, any means that would convert non‐inflammatory apoptosis into a more immunogenic type of cell death might do the trick and improve treatment success. This idea finds support in recent observations documenting that inhibiting caspases after MOMP improves the effects of anti‐cancer therapeutics by promoting an TNF‐driven inflammatory response, activated by proteins released from the mitochondrial inner membrane space, neutralizing inhibitor of apoptosis proteins (IAPs) by promoting their degradation. This leads to NF‐κB‐dependent inflammation, M1 macrophage polarization, and T‐cell‐dependent anti‐tumor immunity 177.

## Conflict of interest

The authors declare that they have no conflict of interest.

**Box 1: In need of answers**

- What is the contribution of cell death to the clinical efficacy of anti‐mitotic drugs?
- Are phosphorylation events detected on different BCL2 proteins during prolonged mitosis relevant for therapy efficacy?
- Is apoptosis the only type of cell death induced in response to anti‐mitotic drugs?
- Do the cell death modalities and/or mediators differ in mitosis vs. death upon mitotic slippage?
- What determines if p53 activation in response to mitotic errors triggers cell death *vs*. cell cycle arrest/senescence?
- Can prolonged mitotic arrest lead to inflammation and what prevents cGAS activation during normal mitosis
- Do extra centrosomes represent a *bona fide* danger signal that can drive inflammation?

## References

[1] Frederiks CN , Lam SW , Guchelaar HJ , Boven E (2015) Genetic polymorphisms and paclitaxel‐ or docetaxel‐induced toxicities: a systematic review. Cancer Treat Rev 41: 935–950 26585358 10.1016/j.ctrv.2015.10.010

[2] Otto T , Sicinski P (2017) Cell cycle proteins as promising targets in cancer therapy. Nat Rev 17: 93–115 10.1038/nrc.2016.138 PMC5345933 28127048

[3] Malumbres M (2011) Physiological relevance of cell cycle kinases. Physiol Rev 91: 973–1007 21742793 10.1152/physrev.00025.2010

[4] Shi J , Mitchison TJ (2017) Cell death response to anti‐mitotic drug treatment in cell culture, mouse tumor model and the clinic. Endocr Relat Cancer 24: T83–T96 28249963 10.1530/ERC-17-0003 PMC5557680

[5] Manchado E , Guillamot M , Malumbres M (2012) Killing cells by targeting mitosis. Cell Death Differ 19: 369–377 22223105 10.1038/cdd.2011.197 PMC3278741

[6] Santaguida S , Amon A (2015) Short‐ and long‐term effects of chromosome mis‐segregation and aneuploidy. Nat Rev Mol Cell Biol 16: 473–485 26204159 10.1038/nrm4025

[7] Orth JD , Loewer A , Lahav G , Mitchison TJ (2012) Prolonged mitotic arrest triggers partial activation of apoptosis, resulting in DNA damage and p53 induction. Mol Biol Cell 23: 567–576 22171325 10.1091/mbc.E11-09-0781 PMC3279386

[8] Uetake Y , Sluder G (2010) Prolonged prometaphase blocks daughter cell proliferation despite normal completion of mitosis. Curr Biol 20: 1666–1671 20832310 10.1016/j.cub.2010.08.018 PMC2946429

[9] Gascoigne KE , Taylor SS (2008) Cancer cells display profound intra‐ and interline variation following prolonged exposure to antimitotic drugs. Cancer Cell 14: 111–122 18656424 10.1016/j.ccr.2008.07.002

[10] Bennett A , Sloss O , Topham C , Nelson L , Tighe A , Taylor SS (2016) Inhibition of Bcl‐xL sensitizes cells to mitotic blockers, but not mitotic drivers. Open Biol 6: 160134 27512141 10.1098/rsob.160134 PMC5008013

[11] Topham C , Tighe A , Ly P , Bennett A , Sloss O , Nelson L , Ridgway RA , Huels D , Littler S , Schandl C *et al* (2015) MYC is a major determinant of mitotic cell fate. Cancer Cell 28: 129–140 26175417 10.1016/j.ccell.2015.06.001 PMC4518499

[12] Topham CH , Taylor SS (2013) Mitosis and apoptosis: how is the balance set? Curr Opin Cell Biol 25: 780–785 23890995 10.1016/j.ceb.2013.07.003

[13] Lopez‐Garcia C , Sansregret L , Domingo E , McGranahan N , Hobor S , BirkBAK1 NJ , Horswell S , Gronroos E , Favero F , Rowan AJ *et al* (2017) BCL9L dysfunction impairs caspase‐2 expression permitting aneuploidy tolerance in colorectal cancer. Cancer Cell 31: 79–93 28073006 10.1016/j.ccell.2016.11.001 PMC5225404

[14] Sheltzer JM , Ko JH , Replogle JM , Habibe Burgos NC , Chung ES , Meehl CM , Sayles NM , Passerini V , Storchova Z , Amon A (2017) Single‐chromosome gains commonly function as tumor suppressors. Cancer Cell 31: 240–255 28089890 10.1016/j.ccell.2016.12.004 PMC5713901

[15] Zasadil LM , Andersen KA , Yeum D , Rocque GB , Wilke LG , Tevaarwerk AJ , Raines RT , Burkard ME , Weaver BA (2014) Cytotoxicity of paclitaxel in breast cancer is due to chromosome missegregation on multipolar spindles. Sci Transl Med 6: 229ra43 10.1126/scitranslmed.3007965 PMC4176609 24670687

[16] Mitchison TJ (2012) The proliferation rate paradox in antimitotic chemotherapy. Mol Biol Cell 23: 1–6 22210845 10.1091/mbc.E10-04-0335 PMC3248889

[17] Galluzzi L , Zitvogel L , Kroemer G (2016) Immunological mechanisms underneath the efficacy of cancer therapy. Cancer Immunol Res 4: 895–902 27803050 10.1158/2326-6066.CIR-16-0197

[18] Harding SM , Benci JL , Irianto J , Discher DE , Minn AJ , Greenberg RA (2017) Mitotic progression following DNA damage enables pattern recognition within micronuclei. Nature 548: 466–470 28759889 10.1038/nature23470 PMC5857357

[19] Lambrus BG , Holland AJ (2017) A new mode of mitotic surveillance. Trends Cell Biol 27: 314–321 28188027 10.1016/j.tcb.2017.01.004 PMC5403546

[20] Sladky V , Schuler F , Fava LL , Villunger A (2017) The resurrection of the PIDDosome ‐ emerging roles in the DNA‐damage response and centrosome surveillance. J Cell Sci 130: 3779–3787 29142064 10.1242/jcs.203448

[21] Musacchio A (2015) The molecular biology of spindle assembly checkpoint signaling dynamics. Curr Biol 25: R1002–R1018 26485365 10.1016/j.cub.2015.08.051

[22] Vigneron S , Prieto S , Bernis C , Labbe JC , Castro A , Lorca T (2004) Kinetochore localization of spindle checkpoint proteins: who controls whom? Mol Biol Cell 15: 4584–4596 15269280 10.1091/mbc.E04-01-0051 PMC519151

[23] Martin‐Lluesma S , Stucke VM , Nigg EA (2002) Role of Hec1 in spindle checkpoint signaling and kinetochore recruitment of Mad1/Mad2. Science 297: 2267–2270 12351790 10.1126/science.1075596

[24] Hiruma Y , Sacristan C , Pachis ST , Adamopoulos A , Kuijt T , Ubbink M , von Castelmur E , Perrakis A , Kops GJ (2015) CELL DIVISION CYCLE. Competition between MPS1 and microtubules at kinetochores regulates spindle checkpoint signaling. Science 348: 1264–1267 26068855 10.1126/science.aaa4055

[25] Ji Z , Gao H , Yu H (2015) CELL DIVISION CYCLE. Kinetochore attachment sensed by competitive Mps1 and microtubule binding to Ndc80C. Science 348: 1260–1264 26068854 10.1126/science.aaa4029

[26] De Antoni A , Pearson CG , Cimini D , Canman JC , Sala V , Nezi L , Mapelli M , Sironi L , Faretta M , Salmon ED *et al* (2005) The Mad1/Mad2 complex as a template for Mad2 activation in the spindle assembly checkpoint. Curr Biol 15: 214–225 15694304 10.1016/j.cub.2005.01.038

[27] Luo X , Tang Z , Xia G , Wassmann K , Matsumoto T , Rizo J , Yu H (2004) The Mad2 spindle checkpoint protein has two distinct natively folded states. Nat Struct Mol Biol 11: 338–345 15024386 10.1038/nsmb748

[28] Luo X , Tang Z , Rizo J , Yu H (2002) The Mad2 spindle checkpoint protein undergoes similar major conformational changes upon binding to either Mad1 or Cdc20. Mol Cell 9: 59–71 11804586 10.1016/s1097-2765(01)00435-x

[29] Mapelli M , Massimiliano L , Santaguida S , Musacchio A (2007) The Mad2 conformational dimer: structure and implications for the spindle assembly checkpoint. Cell 131: 730–743 18022367 10.1016/j.cell.2007.08.049

[30] Hewitt L , Tighe A , Santaguida S , White AM , Jones CD , Musacchio A , Green S , Taylor SS (2010) Sustained Mps1 activity is required in mitosis to recruit O‐Mad2 to the Mad1‐C‐Mad2 core complex. J Cell Biol 190: 25–34 20624899 10.1083/jcb.201002133 PMC2911659

[31] Fava LL , Kaulich M , Nigg EA , Santamaria A (2011) Probing the *in vivo* function of Mad1:C‐Mad2 in the spindle assembly checkpoint. EMBO J 30: 3322–3336 21772247 10.1038/emboj.2011.239 PMC3160659

[32] Han JS , Holland AJ , Fachinetti D , Kulukian A , Cetin B , Cleveland DW (2013) Catalytic assembly of the mitotic checkpoint inhibitor BubR1‐Cdc20 by a Mad2‐induced functional switch in Cdc20. Mol Cell 51: 92–104 23791783 10.1016/j.molcel.2013.05.019 PMC3713096

[33] Faesen AC , Thanasoula M , Maffini S , Breit C , Muller F , van Gerwen S , Bange T , Musacchio A (2017) Basis of catalytic assembly of the mitotic checkpoint complex. Nature 542: 498–502 28102834 10.1038/nature21384 PMC5448665

[34] Herzog F , Primorac I , Dube P , Lenart P , Sander B , Mechtler K , Stark H , Peters JM (2009) Structure of the anaphase‐promoting complex/cyclosome interacting with a mitotic checkpoint complex. Science 323: 1477–1481 19286556 10.1126/science.1163300 PMC2989460

[35] Burton JL , Solomon MJ (2007) Mad3p, a pseudosubstrate inhibitor of APCCdc20 in the spindle assembly checkpoint. Genes Dev 21: 655–667 17369399 10.1101/gad.1511107 PMC1820940

[36] Mansfeld J , Collin P , Collins MO , Choudhary JS , Pines J (2011) APC15 drives the turnover of MCC‐CDC20 to make the spindle assembly checkpoint responsive to kinetochore attachment. Nat Cell Biol 13: 1234–1243 21926987 10.1038/ncb2347 PMC3188299

[37] Izawa D , Pines J (2015) The mitotic checkpoint complex binds a second CDC20 to inhibit active APC/C. Nature 517: 631–634 25383541 10.1038/nature13911 PMC4312099

[38] Howell BJ , McEwen BF , Canman JC , Hoffman DB , Farrar EM , Rieder CL , Salmon ED (2001) Cytoplasmic dynein/dynactin drives kinetochore protein transport to the spindle poles and has a role in mitotic spindle checkpoint inactivation. J Cell Biol 155: 1159–1172 11756470 10.1083/jcb.200105093 PMC2199338

[39] Mapelli M , Filipp FV , Rancati G , Massimiliano L , Nezi L , Stier G , Hagan RS , Confalonieri S , Piatti S , Sattler M *et al* (2006) Determinants of conformational dimerization of Mad2 and its inhibition by p31comet. EMBO J 25: 1273–1284 16525508 10.1038/sj.emboj.7601033 PMC1422169

[40] Yang M , Li B , Tomchick DR , Machius M , Rizo J , Yu H , Luo X (2007) p31comet blocks Mad2 activation through structural mimicry. Cell 131: 744–755 18022368 10.1016/j.cell.2007.08.048 PMC2144745

[41] Teichner A , Eytan E , Sitry‐Shevah D , Miniowitz‐Shemtov S , Dumin E , Gromis J , Hershko A (2011) p31comet Promotes disassembly of the mitotic checkpoint complex in an ATP‐dependent process. Proc Natl Acad Sci USA 108: 3187–3192 21300909 10.1073/pnas.1100023108 PMC3044357

[42] Varetti G , Guida C , Santaguida S , Chiroli E , Musacchio A (2011) Homeostatic control of mitotic arrest. Mol Cell 44: 710–720 22152475 10.1016/j.molcel.2011.11.014

[43] Pinsky BA , Nelson CR , Biggins S (2009) Protein phosphatase 1 regulates exit from the spindle checkpoint in budding yeast. Curr Biol 19: 1182–1187 19592248 10.1016/j.cub.2009.06.043 PMC2731492

[44] Vanoosthuyse V , Hardwick KG (2009) A novel protein phosphatase 1‐dependent spindle checkpoint silencing mechanism. Curr Biol 19: 1176–1181 19592249 10.1016/j.cub.2009.05.060 PMC2791888

[45] Espert A , Uluocak P , Bastos RN , Mangat D , Graab P , Gruneberg U (2014) PP2A‐B56 opposes Mps1 phosphorylation of Knl1 and thereby promotes spindle assembly checkpoint silencing. J Cell Biol 206: 833–842 25246613 10.1083/jcb.201406109 PMC4178970

[46] Yang Z , Kenny AE , Brito DA , Rieder CL (2009) Cells satisfy the mitotic checkpoint in Taxol, and do so faster in concentrations that stabilize syntelic attachments. J Cell Biol 186: 675–684 19720871 10.1083/jcb.200906150 PMC2742195

[47] Mayer TU , Kapoor TM , Haggarty SJ , King RW , Schreiber SL , Mitchison TJ (1999) Small molecule inhibitor of mitotic spindle bipolarity identified in a phenotype‐based screen. Science 286: 971–974 10542155 10.1126/science.286.5441.971

[48] Brito DA , Rieder CL (2006) Mitotic checkpoint slippage in humans occurs via cyclin B destruction in the presence of an active checkpoint. Curr Biol 16: 1194–1200 16782009 10.1016/j.cub.2006.04.043 PMC2749311

[49] Collin P , Nashchekina O , Walker R , Pines J (2013) The spindle assembly checkpoint works like a rheostat rather than a toggle switch. Nat Cell Biol 15: 1378–1385 24096242 10.1038/ncb2855 PMC3836401

[50] Diaz‐Martinez LA , Karamysheva ZN , Warrington R , Li B , Wei S , Xie XJ , Roth MG , Yu H (2014) Genome‐wide siRNA screen reveals coupling between mitotic apoptosis and adaptation. EMBO J 33: 1960–1976 25024437 10.15252/embj.201487826 PMC4195789

[51] Huang HC , Mitchison TJ , Shi J (2010) Stochastic competition between mechanistically independent slippage and death pathways determines cell fate during mitotic arrest. PLoS ONE 5: e15724 21203573 10.1371/journal.pone.0015724 PMC3006339

[52] Haschka MD , Soratroi C , Kirschnek S , Hacker G , Hilbe R , Geley S , Villunger A , Fava LL (2015) The NOXA‐MCL1‐BIM axis defines lifespan on extended mitotic arrest. Nat Commun 6: 6891 25922916 10.1038/ncomms7891 PMC4423218

[53] Domenech E , Maestre C , Esteban‐Martinez L , Partida D , Pascual R , Fernandez‐Miranda G , Seco E , Campos‐Olivas R , Perez M , Megias D *et al* (2015) AMPK and PFKFB3 mediate glycolysis and survival in response to mitophagy during mitotic arrest. Nat Cell Biol 17: 1304–1316 26322680 10.1038/ncb3231

[54] Sloss O , Topham C , Diez M , Taylor S (2016) Mcl‐1 dynamics influence mitotic slippage and death in mitosis. Oncotarget 7: 5176–5192 26769847 10.18632/oncotarget.6894 PMC4868679

[55] Huang HC , Shi J , Orth JD , Mitchison TJ (2009) Evidence that mitotic exit is a better cancer therapeutic target than spindle assembly. Cancer Cell 16: 347–358 19800579 10.1016/j.ccr.2009.08.020 PMC2758291

[56] Zeng X , Sigoillot F , Gaur S , Choi S , Pfaff KL , Oh DC , Hathaway N , Dimova N , Cuny GD , King RW (2010) Pharmacologic inhibition of the anaphase‐promoting complex induces a spindle checkpoint‐dependent mitotic arrest in the absence of spindle damage. Cancer Cell 18: 382–395 20951947 10.1016/j.ccr.2010.08.010 PMC2957475

[57] Fong CS , Mazo G , Das T , Goodman J , Kim M , O'Rourke BP , Izquierdo D , Tsou MF (2016) 53BP1 and USP28 mediate p53‐dependent cell cycle arrest in response to centrosome loss and prolonged mitosis. eLife 5: e16270 27371829 10.7554/eLife.16270 PMC4946878

[58] Lambrus BG , Daggubati V , Uetake Y , Scott PM , Clutario KM , Sluder G , Holland AJ (2016) A USP28‐53BP1‐p53‐p21 signaling axis arrests growth after centrosome loss or prolonged mitosis. J Cell Biol 214: 143–153 27432896 10.1083/jcb.201604054 PMC4949452

[59] Meitinger F , Anzola JV , Kaulich M , Richardson A , Stender JD , Benner C , Glass CK , Dowdy SF , Desai A , Shiau AK *et al* (2016) 53BP1 and USP28 mediate p53 activation and G1 arrest after centrosome loss or extended mitotic duration. J Cell Biol 214: 155–166 27432897 10.1083/jcb.201604081 PMC4949453

[60] Hinchcliffe EH , Day CA , Karanjeet KB , Fadness S , Langfald A , Vaughan KT , Dong Z (2016) Chromosome missegregation during anaphase triggers p53 cell cycle arrest through histone H3.3 Ser31 phosphorylation. Nat Cell Biol 18: 668–675 27136267 10.1038/ncb3348

[61] Thompson SL , Compton DA (2010) Proliferation of aneuploid human cells is limited by a p53‐dependent mechanism. J Cell Biol 188: 369–381 20123995 10.1083/jcb.200905057 PMC2819684

[62] Palozola KC , Donahue G , Liu H , Grant GR , Becker JS , Cote A , Yu H , Raj A , Zaret KS (2017) Mitotic transcription and waves of gene reactivation during mitotic exit. Science 358: 119–122 28912132 10.1126/science.aal4671 PMC5727891

[63] Michalak E , Villunger A , Erlacher M , Strasser A (2005) Death squads enlisted by the tumour suppressor p53. Biochem Biophys Res Commun 331: 786–798 15865934 10.1016/j.bbrc.2005.03.183

[64] Woo M , Hakem R , Furlonger C , Hakem A , Duncan GS , Sasaki T , Bouchard D , Lu L , Wu GE , Paige CJ *et al* (2003) Caspase‐3 regulates cell cycle in B cells: a consequence of substrate specificity. Nat Immunol 4: 1016–1022 12970760 10.1038/ni976

[65] Podmirseg SR , Jakel H , Ranches GD , Kullmann MK , Sohm B , Villunger A , Lindner H , Hengst L (2016) Caspases uncouple p27(Kip1) from cell cycle regulated degradation and abolish its ability to stimulate cell migration and invasion. Oncogene 35: 4580–4590 26829051 10.1038/onc.2015.524 PMC4854979

[66] Craxton A , Draves KE , Clark EA (2007) Bim regulates BCR‐induced entry of B cells into the cell cycle. Eur J Immunol 37: 2715–2722 17705137 10.1002/eji.200737327

[67] Ichim G , Lopez J , Ahmed SU , Muthalagu N , Giampazolias E , Delgado ME , Haller M , Riley JS , Mason SM , Athineos D *et al* (2015) Limited mitochondrial permeabilization causes DNA damage and genomic instability in the absence of cell death. Mol Cell 57: 860–872 25702873 10.1016/j.molcel.2015.01.018 PMC4352766

[68] Deng X , Gao F , Flagg T , May WS Jr (2004) Mono‐ and multisite phosphorylation enhances Bcl2's antiapoptotic function and inhibition of cell cycle entry functions. Proc Natl Acad Sci USA 101: 153–158 14660795 10.1073/pnas.2533920100 PMC314154

[69] Vairo G , Soos TJ , Upton TM , Zalvide J , DeCaprio JA , Ewen ME , Koff A , Adams JM (2000) Bcl‐2 retards cell entry through p27^Kip1^, pRB relative p130 and altered E2F regulation. Mol Cell Biol 20: 4745–4753 10848600 10.1128/mcb.20.13.4745-4753.2000 PMC85901

[70] Crasta K , Ganem NJ , Dagher R , Lantermann AB , Ivanova EV , Pan Y , Nezi L , Protopopov A , Chowdhury D , Pellman D (2012) DNA breaks and chromosome pulverization from errors in mitosis. Nature 482: 53–58 22258507 10.1038/nature10802 PMC3271137

[71] Ito Y , Hoare M , Narita M (2017) Spatial and temporal control of senescence. Trends Cell Biol 27: 820–832 28822679 10.1016/j.tcb.2017.07.004

[72] Fava LL , Schuler F , Sladky V , Haschka MD , Soratroi C , Eiterer L , Demetz E , Weiss G , Geley S , Nigg EA *et al* (2017) The PIDDosome activates p53 in response to supernumerary centrosomes. Genes Dev 31: 34–45 28130345 10.1101/gad.289728.116 PMC5287111

[73] Tuzlak S , Kaufmann T , Villunger A (2016) Interrogating the relevance of mitochondrial apoptosis for vertebrate development and postnatal tissue homeostasis. Genes Dev 30: 2133–2151 27798841 10.1101/gad.289298.116 PMC5088563

[74] Czabotar PE , Lessene G , Strasser A , Adams JM (2014) Control of apoptosis by the BCL‐2 protein family: implications for physiology and therapy. Nat Rev Mol Cell Biol 15: 49–63 24355989 10.1038/nrm3722

[75] Salvador‐Gallego R , Mund M , Cosentino K , Schneider J , Unsay J , Schraermeyer U , Engelhardt J , Ries J , Garcia‐Saez AJ (2016) Bax assembly into rings and arcs in apoptotic mitochondria is linked to membrane pores. EMBO J 35: 389–401 26783362 10.15252/embj.201593384 PMC4755116

[76] Bleicken S , Jeschke G , Stegmueller C , Salvador‐Gallego R , Garcia‐Saez AJ , Bordignon E (2014) Structural model of active Bax at the membrane. Mol Cell 56: 496–505 25458844 10.1016/j.molcel.2014.09.022 PMC4869853

[77] Barille‐Nion S , Bah N , Vequaud E , Juin P (2012) Regulation of cancer cell survival by BCL2 family members upon prolonged mitotic arrest: opportunities for anticancer therapy. Anticancer Res 32: 4225–4233 23060542

[78] Panopoulos A , Pacios‐Bras C , Choi J , Yenjerla M , Sussman MA , Fotedar R , Margolis RL (2014) Failure of cell cleavage induces senescence in tetraploid primary cells. Mol Biol Cell 25: 3105–3118 25143403 10.1091/mbc.E14-03-0844 PMC4196863

[79] De Santis Puzzonia M , Gonzalez L , Ascenzi S , Cundari E , Degrassi F (2016) Tetraploid cells produced by absence of substrate adhesion during cytokinesis are limited in their proliferation and enter senescence after DNA replication. Cell Cycle 15: 274–282 26693937 10.1080/15384101.2015.1127469 PMC4825921

[80] O'Neill KL , Huang K , Zhang J , Chen Y , Luo X (2016) Inactivation of prosurvival Bcl‐2 proteins activates Bax/BAK1 through the outer mitochondrial membrane. Genes Dev 30: 973–988 27056669 10.1101/gad.276725.115 PMC4840302

[81] Shi J , Zhou Y , Huang HC , Mitchison TJ (2011) Navitoclax (ABT‐263) accelerates apoptosis during drug‐induced mitotic arrest by antagonizing Bcl‐xL. Can Res 71: 4518–4526 10.1158/0008-5472.CAN-10-4336 PMC3129452 21546570

[82] Huang S , Tang R , Poon RY (2016) BCL‐W is a regulator of microtubule inhibitor‐induced mitotic cell death. Oncotarget 7: 38718–38730 27231850 10.18632/oncotarget.9586 PMC5122423

[83] Tan TT , Degenhardt K , Nelson DA , Beaudoin B , Nieves‐Neira W , Bouillet P , Villunger A , Adams JM , White E (2005) Key roles of BIM‐driven apoptosis in epithelial tumors and rational chemotherapy. Cancer Cell 7: 227–238 15766661 10.1016/j.ccr.2005.02.008

[84] Gao Z , Shang Q , Liu Z , Deng C , Guo C (2015) Mitochondrial ATF2 translocation contributes to apoptosis induction and BRAF inhibitor resistance in melanoma through the interaction of Bim with VDAC1. Oncotarget 6: 36338–36353 26462148 10.18632/oncotarget.5537 PMC4742181

[85] Wang P , Lindsay J , Owens TW , Mularczyk EJ , Warwood S , Foster F , Streuli CH , Brennan K , Gilmore AP (2014) Phosphorylation of the proapoptotic BH3‐only protein bid primes mitochondria for apoptosis during mitotic arrest. Cell Rep 7: 661–671 24767991 10.1016/j.celrep.2014.03.050 PMC4022835

[86] Shah OJ , Lin X , Li L , Huang X , Li J , Anderson MG , Tang H , Rodriguez LE , Warder SE , McLoughlin S *et al* (2010) Bcl‐XL represents a druggable molecular vulnerability during aurora B inhibitor‐mediated polyploidization. Proc Natl Acad Sci USA 107: 12634–12639 20616035 10.1073/pnas.0913615107 PMC2906553

[87] Erlacher M , Labi V , Manzl C , Bock G , Tzankov A , Hacker G , Michalak E , Strasser A , Villunger A (2006) Puma cooperates with Bim, the rate‐limiting BH3‐only protein in cell death during lymphocyte development, in apoptosis induction. J Exp Med 203: 2939–2951 17178918 10.1084/jem.20061552 PMC2118188

[88] Labi V , Woess C , Tuzlak S , Erlacher M , Bouillet P , Strasser A , Tzankov A , Villunger A (2014) Deregulated cell death and lymphocyte homeostasis cause premature lethality in mice lacking the BH3‐only proteins Bim and Bmf. Blood 123: 2652–2662 24632712 10.1182/blood-2013-11-537217 PMC3999752

[89] Bah N , Maillet L , Ryan J , Dubreil S , Gautier F , Letai A , Juin P , Barille‐Nion S (2014) Bcl‐xL controls a switch between cell death modes during mitotic arrest. Cell Death Dis 5: e1291 24922075 10.1038/cddis.2014.251 PMC4611724

[90] Kutuk O , Letai A (2010) Displacement of Bim by Bmf and Puma rather than increase in Bim level mediates paclitaxel‐induced apoptosis in breast cancer cells. Cell Death Differ 17: 1624–1635 20431602 10.1038/cdd.2010.41 PMC2914832

[91] Souers AJ , Leverson JD , Boghaert ER , Ackler SL , Catron ND , Chen J , Dayton BD , Ding H , Enschede SH , Fairbrother WJ *et al* (2013) ABT‐199, a potent and selective BCL‐2 inhibitor, achieves antitumor activity while sparing platelets. Nat Med 19: 202–208 23291630 10.1038/nm.3048

[92] Konopleva M , Pollyea DA , Potluri J , Chyla B , Hogdal L , Busman T , McKeegan E , Salem AH , Zhu M , Ricker JL *et al* (2016) Efficacy and biological correlates of response in a phase ii study of venetoclax monotherapy in patients with acute myelogenous leukemia. Cancer Discov 6: 1106–1117 27520294 10.1158/2159-8290.CD-16-0313 PMC5436271

[93] Dai H , Ding H , Meng XW , Lee SH , Schneider PA , Kaufmann SH (2013) Contribution of Bcl‐2 phosphorylation to BAK1 binding and drug resistance. Can Res 73: 6998–7008 10.1158/0008-5472.CAN-13-0940 PMC3910374 24097825

[94] Gilot D , Serandour AL , Ilyin GP , Lagadic‐Gossmann D , Loyer P , Corlu A , Coutant A , Baffet G , Peter ME , Fardel O *et al* (2005) A role for caspase‐8 and c‐FLIPL in proliferation and cell‐cycle progression of primary hepatocytes. Carcinogenesis 26: 2086–2094 16033771 10.1093/carcin/bgi187

[95] Alappat EC , Feig C , Boyerinas B , Volkland J , Samuels M , Murmann AE , Thorburn A , Kidd VJ , Slaughter CA , Osborn SL *et al* (2005) Phosphorylation of FADD at serine 194 by CKIalpha regulates its nonapoptotic activities. Mol Cell 19: 321–332 16061179 10.1016/j.molcel.2005.06.024

[96] van Raam BJ , Salvesen GS (2012) Proliferative versus apoptotic functions of caspase‐8 Hetero or homo: the caspase‐8 dimer controls cell fate. Biochem Biophys Acta 1824: 113–122 21704196 10.1016/j.bbapap.2011.06.005 PMC3993904

[97] Castedo M , Perfettini JL , Roumier T , Valent A , Raslova H , Yakushijin K , Horne D , Feunteun J , Lenoir G , Medema R *et al* (2004) Mitotic catastrophe constitutes a special case of apoptosis whose suppression entails aneuploidy. Oncogene 23: 4362–4370 15048075 10.1038/sj.onc.1207572

[98] Ho LH , Read SH , Dorstyn L , Lambrusco L , Kumar S (2008) Caspase‐2 is required for cell death induced by cytoskeletal disruption. Oncogene 27: 3393–3404 18193089 10.1038/sj.onc.1211005

[99] Manzl C , Krumschnabel G , Bock F , Sohm B , Labi V , Baumgartner F , Logette E , Tschopp J , Villunger A (2009) Caspase‐2 activation in the absence of PIDDosome formation. J Cell Biol 185: 291–303 19364921 10.1083/jcb.200811105 PMC2700374

[100] Gao Z , Shao Y , Jiang X (2005) Essential roles of the Bcl‐2 family of proteins in caspase‐2‐induced apoptosis. J Biol Chem 280: 38271–38275 16172118 10.1074/jbc.M506488200

[101] Scatena CD , Stewart ZA , Mays D , Tang LJ , Keefer CJ , Leach SD , Pietenpol JA (1998) Mitotic phosphorylation of Bcl‐2 during normal cell cycle progression and Taxol‐induced growth arrest. J Biol Chem 273: 30777–30784 9804855 10.1074/jbc.273.46.30777

[102] Yamamoto K , Ichijo H , Korsmeyer SJ (1999) BCL‐2 Is Phosphorylated and Inactivated by an ASK1/Jun N‐Terminal Protein Kinase Pathway Normally Activated at G_2_/M. Mol Cell Biol 19: 8469–8478 10567572 10.1128/mcb.19.12.8469 PMC84954

[103] Han CR , Jun do Y , Lee JY , Kim YH (2014) Prometaphase arrest‐dependent phosphorylation of Bcl‐2 and Bim reduces the association of Bcl‐2 with BAK1 or Bim, provoking BAK1 activation and mitochondrial apoptosis in nocodazole‐treated Jurkat T cells. Apoptosis 19: 224–240 24166139 10.1007/s10495-013-0928-1

[104] Eichhorn JM , Sakurikar N , Alford SE , Chu R , Chambers TC (2013) Critical role of anti‐apoptotic Bcl‐2 protein phosphorylation in mitotic death. Cell Death Dis 4: e834 24091677 10.1038/cddis.2013.360 PMC3824670

[105] Zhou L , Cai X , Han X , Xu N , Chang DC (2014) CDK1 switches mitotic arrest to apoptosis by phosphorylating Bcl‐2/Bax family proteins during treatment with microtubule interfering agents. Cell Biol Int 38: 737–746 24677263 10.1002/cbin.10259

[106] Brichese L , Barboule N , Heliez C , Valette A (2002) Bcl‐2 phosphorylation and proteasome‐dependent degradation induced by paclitaxel treatment: consequences on sensitivity of isolated mitochondria to Bid. Exp Cell Res 278: 101–111 12126962 10.1006/excr.2002.5563

[107] Brichese L , Valette A (2002) PP1 phosphatase is involved in Bcl‐2 dephosphorylation after prolonged mitotic arrest induced by paclitaxel. Biochem Biophys Res Commun 294: 504–508 12051739 10.1016/S0006-291X(02)00505-3

[108] Choi HJ , Zhu BT (2014) Role of cyclin B1/Cdc2 in mediating Bcl‐XL phosphorylation and apoptotic cell death following nocodazole‐induced mitotic arrest. Mol Carcinog 53: 125–137 22949227 10.1002/mc.21956

[109] Wei Y , Pattingre S , Sinha S , Bassik M , Levine B (2008) JNK1‐mediated phosphorylation of Bcl‐2 regulates starvation‐induced autophagy. Mol Cell 30: 678–688 18570871 10.1016/j.molcel.2008.06.001 PMC2478643

[110] Wei Y , Sinha S , Levine B (2008) Dual role of JNK1‐mediated phosphorylation of Bcl‐2 in autophagy and apoptosis regulation. Autophagy 4: 949–951 18769111 10.4161/auto.6788 PMC2677707

[111] Du L , Lyle CS , Chambers TC (2005) Characterization of vinblastine‐induced Bcl‐xL and Bcl‐2 phosphorylation: evidence for a novel protein kinase and a coordinated phosphorylation/dephosphorylation cycle associated with apoptosis induction. Oncogene 24: 107–117 15531923 10.1038/sj.onc.1208189

[112] Upreti M , Lyle CS , Skaug B , Du L , Chambers TC (2006) Vinblastine‐induced apoptosis is mediated by discrete alterations in subcellular location, oligomeric structure, and activation status of specific Bcl‐2 family members. J Biol Chem 281: 15941–15950 16574665 10.1074/jbc.M512586200 PMC1656399

[113] Terrano DT , Upreti M , Chambers TC (2010) Cyclin‐dependent kinase 1‐mediated Bcl‐xL/Bcl‐2 phosphorylation acts as a functional link coupling mitotic arrest and apoptosis. Mol Cell Biol 30: 640–656 19917720 10.1128/MCB.00882-09 PMC2812246

[114] Wang J , Beauchemin M , Bertrand R (2011) Bcl‐xL phosphorylation at Ser49 by polo kinase 3 during cell cycle progression and checkpoints. Cell Signal 23: 2030–2038 21840391 10.1016/j.cellsig.2011.07.017 PMC3708862

[115] Baruah PS , Beauchemin M , Hebert J , Bertrand R (2016) Dynamic Bcl‐xL (S49) and (S62) phosphorylation/dephosphorylation during mitosis prevents chromosome instability and aneuploidy in normal human diploid fibroblasts. PLoS ONE 11: e0159091 27398719 10.1371/journal.pone.0159091 PMC4939973

[116] Sochalska M , Tuzlak S , Egle A , Villunger A (2015) Lessons from gain‐ and loss‐of‐function models of pro‐survival Bcl2 family proteins: implications for targeted therapy. FEBS J 282: 834–849 25559680 10.1111/febs.13188 PMC4562365

[117] Harley ME , Allan LA , Sanderson HS , Clarke PR (2010) Phosphorylation of Mcl‐1 by CDK1‐cyclin B1 initiates its Cdc20‐dependent destruction during mitotic arrest. EMBO J 29: 2407–2420 20526282 10.1038/emboj.2010.112 PMC2910263

[118] Wertz IE , Kusam S , Lam C , Okamoto T , Sandoval W , Anderson DJ , Helgason E , Ernst JA , Eby M , Liu J *et al* (2011) Sensitivity to antitubulin chemotherapeutics is regulated by MCL1 and FBW7. Nature 471: 110–114 21368834 10.1038/nature09779

[119] Inuzuka H , Shaik S , Onoyama I , Gao D , Tseng A , Maser RS , Zhai B , Wan L , Gutierrez A , Lau AW *et al* (2011) SCF(FBW7) regulates cellular apoptosis by targeting MCL1 for ubiquitylation and destruction. Nature 471: 104–109 21368833 10.1038/nature09732 PMC3076007

[120] Chiu WH , Luo SJ , Chen CL , Cheng JH , Hsieh CY , Wang CY , Huang WC , Su WC , Lin CF (2012) Vinca alkaloids cause aberrant ROS‐mediated JNK activation, Mcl‐1 downregulation, DNA damage, mitochondrial dysfunction, and apoptosis in lung adenocarcinoma cells. Biochem Pharmacol 83: 1159–1171 22285910 10.1016/j.bcp.2012.01.016

[121] Chu R , Terrano DT , Chambers TC (2012) Cdk1/cyclin B plays a key role in mitotic arrest‐induced apoptosis by phosphorylation of Mcl‐1, promoting its degradation and freeing BAK1 from sequestration. Biochem Pharmacol 83: 199–206 22024133 10.1016/j.bcp.2011.10.008 PMC3242896

[122] Chu R , Alford SE , Hart K , Kothari A , Mackintosh SG , Kovak MR , Chambers TC (2016) Mitotic arrest‐induced phosphorylation of Mcl‐1 revisited using two‐dimensional gel electrophoresis and phosphoproteomics: nine phosphorylation sites identified. Oncotarget 7: 78958–78970 27738316 10.18632/oncotarget.12586 PMC5346690

[123] Stewart DP , Koss B , Bathina M , Perciavalle RM , Bisanz K , Opferman JT (2010) Ubiquitin‐independent degradation of antiapoptotic MCL‐1. Mol Cell Biol 30: 3099–3110 20385764 10.1128/MCB.01266-09 PMC2876674

[124] Craxton A , Butterworth M , Harper N , Fairall L , Schwabe J , Ciechanover A , Cohen GM (2012) NOXA, a sensor of proteasome integrity, is degraded by 26S proteasomes by an ubiquitin‐independent pathway that is blocked by MCL‐1. Cell Death Differ 19: 1424–1434 22361683 10.1038/cdd.2012.16 PMC3422467

[125] Czabotar PE , Lee EF , van Delft MF , Day CL , Smith BJ , Huang DC , Fairlie WD , Hinds MG , Colman PM (2007) Structural insights into the degradation of Mcl‐1 induced by BH3 domains. Proc Natl Acad Sci USA 104: 6217–6222 17389404 10.1073/pnas.0701297104 PMC1851040

[126] Sugio A , Iwasaki M , Habata S , Mariya T , Suzuki M , Osogami H , Tamate M , Tanaka R , Saito T (2014) BAG3 upregulates Mcl‐1 through downregulation of miR‐29b to induce anticancer drug resistance in ovarian cancer. Gynecol Oncol 134: 615–623 24992675 10.1016/j.ygyno.2014.06.024

[127] Konno Y , Dong P , Xiong Y , Suzuki F , Lu J , Cai M , Watari H , Mitamura T , Hosaka M , Hanley SJ *et al* (2014) MicroRNA‐101 targets EZH2, MCL‐1 and FOS to suppress proliferation, invasion and stem cell‐like phenotype of aggressive endometrial cancer cells. Oncotarget 5: 6049–6062 25153722 10.18632/oncotarget.2157 PMC4171612

[128] Lu C , Xie Z , Peng Q (2017) MiRNA‐107 enhances chemosensitivity to paclitaxel by targeting antiapoptotic factor Bcl‐w in non small cell lung cancer. Am J Cancer Res 7: 1863–1873 28979809 PMC5622221

[129] Herold MJ , Zeitz J , Pelzer C , Kraus C , Peters A , Wohlleben G , Berberich I (2006) The stability and anti‐apoptotic function of A1 are controlled by its C terminus. J Biol Chem 281: 13663–13671 16551634 10.1074/jbc.M600266200

[130] Fan G , Simmons MJ , Ge S , Dutta‐Simmons J , Kucharczak J , Ron Y , Weissmann D , Chen CC , Mukherjee C , White E *et al* (2011) Defective ubiquitin‐mediated degradation of antiapoptotic Bfl‐1 predisposes to lymphoma. Blood 115: 3559–3569 10.1182/blood-2009-08-236760 PMC2867266 20185581

[131] Ottina E , Tischner D , Herold MJ , Villunger A (2012) A1/Bfl‐1 in leukocyte development and cell death. Exp Cell Res 318: 1291–1303 22342458 10.1016/j.yexcr.2012.01.021 PMC3405526

[132] Brien G , Trescol‐Biemont MC , Bonnefoy‐Berard N (2007) Downregulation of Bfl‐1 protein expression sensitizes malignant B cells to apoptosis. Oncogene 26: 5828–5832 17353899 10.1038/sj.onc.1210363

[133] Xia L , Wurmbach E , Waxman S , Jing Y (2006) Upregulation of Bfl‐1/A1 in leukemia cells undergoing differentiation by all‐trans retinoic acid treatment attenuates chemotherapeutic agent‐induced apoptosis. Leukemia 20: 1009–1016 16572199 10.1038/sj.leu.2404198

[134] Upreti M , Chu R , Galitovskaya E , Smart SK , Chambers TC (2008) Key role for BAK1 activation and BAK1‐Bax interaction in the apoptotic response to vinblastine. Mol Cancer Ther 7: 2224–2232 18645031 10.1158/1535-7163.MCT-07-2299 PMC3276366

[135] Miller AV , Hicks MA , Nakajima W , Richardson AC , Windle JJ , Harada H (2013) Paclitaxel‐induced apoptosis is BAK1‐dependent, but BAX and BIM‐independent in breast tumor. PLoS ONE 8: e60685 23577147 10.1371/journal.pone.0060685 PMC3618047

[136] Gardai SJ , Hildeman DA , Frankel SK , Whitlock BB , Frasch SC , Borregaard N , Marrack P , Bratton DL , Henson PM (2004) Phosphorylation of Bax Ser184 by Akt regulates its activity and apoptosis in neutrophils. J Biol Chem 279: 21085–21095 14766748 10.1074/jbc.M400063200

[137] Fox JL , Storey A (2015) BMX negatively regulates BAK1 function, thereby increasing apoptotic resistance to chemotherapeutic drugs. Can Res 75: 1345–1355 10.1158/0008-5472.CAN-14-1340 PMC4384990 25649765

[138] Fox JL , Ismail F , Azad A , Ternette N , Leverrier S , Edelmann MJ , Kessler BM , Leigh IM , Jackson S , Storey A (2010) Tyrosine dephosphorylation is required for BAK1 activation in apoptosis. EMBO J 29: 3853–3868 20959805 10.1038/emboj.2010.244 PMC2989102

[139] Czernick M , Rieger A , Goping IS (2009) Bim is reversibly phosphorylated but plays a limited role in paclitaxel cytotoxicity of breast cancer cell lines. Biochem Biophys Res Commun 379: 145–150 19101511 10.1016/j.bbrc.2008.12.025

[140] Moustafa‐Kamal M , Gamache I , Lu Y , Li S , Teodoro JG (2013) BimEL is phosphorylated at mitosis by Aurora A and targeted for degradation by betaTrCP1. Cell Death Differ 20: 1393–1403 23912711 10.1038/cdd.2013.93 PMC3770328

[141] Fava LL , Haschka MD , Villunger A (2013) Bim vanishes in the light of a mitotic Aurora. Cell Death Differ 20: 1597–1598 24212928 10.1038/cdd.2013.140 PMC3824599

[142] Wan L , Tan M , Yang J , Inuzuka H , Dai X , Wu T , Liu J , Shaik S , Chen G , Deng J *et al* (2014) APC(Cdc20) suppresses apoptosis through targeting bim for ubiquitination and destruction. Dev Cell 29: 377–391 24871945 10.1016/j.devcel.2014.04.022 PMC4081014

[143] Gilley R , Lochhead PA , Balmanno K , Oxley D , Clark J , Cook SJ (2012) CDK1, not ERK1/2 or ERK5, is required for mitotic phosphorylation of BIMEL. Cell Signal 24: 170–180 21924351 10.1016/j.cellsig.2011.08.018

[144] Sunters A , Fernandez de Mattos S , Stahl M , Brosens JJ , Zoumpoulidou G , Saunders CA , Coffer PJ , Medema RH , Coombes RC , Lam EW (2003) FoxO3a transcriptional regulation of Bim controls apoptosis in paclitaxel‐treated breast cancer cell lines. J Biol Chem 278: 49795–49805 14527951 10.1074/jbc.M309523200

[145] Kawabata T , Tanimura S , Asai K , Kawasaki R , Matsumaru Y , Kohno M (2012) Up‐regulation of pro‐apoptotic protein Bim and down‐regulation of anti‐apoptotic protein Mcl‐1 cooperatively mediate enhanced tumor cell death induced by the combination of ERK kinase (MEK) inhibitor and microtubule inhibitor. J Biol Chem 287: 10289–10300 22270368 10.1074/jbc.M111.319426 PMC3322996

[146] Lowman XH , McDonnell MA , Kosloske A , Odumade OA , Jenness C , Karim CB , Jemmerson R , Kelekar A (2010) The proapoptotic function of Noxa in human leukemia cells is regulated by the kinase Cdk5 and by glucose. Mol Cell 40: 823–833 21145489 10.1016/j.molcel.2010.11.035

[147] Weber A , Heinlein M , Dengjel J , Alber C , Singh PK , Hacker G (2016) The deubiquitinase Usp27x stabilizes the BH3‐only protein Bim and enhances apoptosis. EMBO Rep 17: 724–738 27013495 10.15252/embr.201541392 PMC5341510

[148] Brinkmann K , Zigrino P , Witt A , Schell M , Ackermann L , Broxtermann P , Schull S , Andree M , Coutelle O , Yazdanpanah B *et al* (2013) Ubiquitin C‐terminal hydrolase‐L1 potentiates cancer chemosensitivity by stabilizing NOXA. Cell Rep 3: 881–891 23499448 10.1016/j.celrep.2013.02.014

[149] Schwickart M , Huang X , Lill JR , Liu J , Ferrando R , French DM , Maecker H , O'Rourke K , Bazan F , Eastham‐Anderson J *et al* (2010) Deubiquitinase USP9X stabilizes MCL1 and promotes tumour cell survival. Nature 463: 103–107 20023629 10.1038/nature08646

[150] Czabotar PE , Lessene G , Strasser A , Adams JM (2013) Control of apoptosis by the BCL‐2 protein family: implications for physiology and therapy. Nat Rev Mol Cell Biol 15: 49–63 10.1038/nrm3722 24355989

[151] Berndtsson M , Konishi Y , Bonni A , Hagg M , Shoshan M , Linder S , Havelka AM (2005) Phosphorylation of BAD at Ser‐128 during mitosis and paclitaxel‐induced apoptosis. FEBS Lett 579: 3090–3094 15907327 10.1016/j.febslet.2005.04.067

[152] Danial NN (2008) BAD: undertaker by night, candyman by day. Oncogene 27(Suppl. 1): S53–S70 19641507 10.1038/onc.2009.44

[153] Germain M , Mathai JP , McBride HM , Shore GC (2005) Endoplasmic reticulum BIK initiates DRP1‐regulated remodelling of mitochondrial cristae during apoptosis. EMBO J 24: 1546–1556 15791210 10.1038/sj.emboj.7600592 PMC1142564

[154] Coultas L , Terzano S , Thomas T , Voss A , Reid K , Stanley EG , Scott CL , Bouillet P , Bartlett P , Ham J *et al* (2007) Hrk/DP5 contributes to the apoptosis of select neuronal populations but is dispensable for haematopoietic cell apoptosis. J Cell Sci 120: 2044–2052 17535852 10.1242/jcs.002063 PMC2795636

[155] Janssen A , van der Burg M , Szuhai K , Kops GJ , Medema RH (2011) Chromosome segregation errors as a cause of DNA damage and structural chromosome aberrations. Science 333: 1895–1898 21960636 10.1126/science.1210214

[156] McCool KW , Miyamoto S (2012) DNA damage‐dependent NF‐kappaB activation: NEMO turns nuclear signaling inside out. Immunol Rev 246: 311–326 22435563 10.1111/j.1600-065X.2012.01101.x PMC3311051

[157] Biton S , Ashkenazi A (2011) NEMO and RIP1 control cell fate in response to extensive DNA damage via TNF‐alpha feedforward signaling. Cell 145: 92–103 21458669 10.1016/j.cell.2011.02.023

[158] Hinz M , Stilmann M , Arslan SC , Khanna KK , Dittmar G , Scheidereit C (2010) A cytoplasmic ATM‐TRAF6‐cIAP1 module links nuclear DNA damage signaling to ubiquitin‐mediated NF‐kappaB activation. Mol Cell 40: 63–74 20932475 10.1016/j.molcel.2010.09.008

[159] Stilmann M , Hinz M , Arslan SC , Zimmer A , Schreiber V , Scheidereit C (2009) A nuclear poly(ADP‐ribose)‐dependent signalosome confers DNA damage‐induced IkappaB kinase activation. Mol Cell 36: 365–378 19917246 10.1016/j.molcel.2009.09.032

[160] Janssens S , Tinel A , Lippens S , Tschopp J (2005) PIDD mediates NF‐kappaB activation in response to DNA damage. Cell 123: 1079–1092 16360037 10.1016/j.cell.2005.09.036

[161] Bock FJ , Krumschnabel G , Manzl C , Peintner L , Tanzer MC , Hermann‐Kleiter N , Baier G , Llacuna L , Yelamos J , Villunger A (2013) Loss of PIDD limits NF‐kappaB activation and cytokine production but not cell survival or transformation after DNA damage. Cell Death Differ 20: 546–557 23238565 10.1038/cdd.2012.152 PMC3595480

[162] Hemmi H , Takeuchi O , Kawai T , Kaisho T , Sato S , Sanjo H , Matsumoto M , Hoshino K , Wagner H , Takeda K *et al* (2000) A Toll‐like receptor recognizes bacterial DNA. Nature 408: 740–745 11130078 10.1038/35047123

[163] Ablasser A , Hornung V (2013) DNA sensing unchained. Cell Res 23: 585–587 23419517 10.1038/cr.2013.28 PMC3641596

[164] Zierhut C , Funabiki H (2017) The cytoplasmic DNA sensor cGAS promotes mitotic cell death. bioRxiv https://doi.org/10.1101/168070 [PREPRINT] 10.1016/j.cell.2019.05.035 PMC6693521 31299200

[165] Mackenzie KJ , Carroll P , Martin CA , Murina O , Fluteau A , Simpson DJ , Olova N , Sutcliffe H , Rainger JK , Leitch A *et al* (2017) cGAS surveillance of micronuclei links genome instability to innate immunity. Nature 548: 461–465 28738408 10.1038/nature23449 PMC5870830

[166] Bartsch K , Knittler K , Borowski C , Rudnik S , Damme M , Aden K , Spehlmann ME , Frey N , Saftig P , Chalaris A *et al* (2017) Absence of RNase H2 triggers generation of immunogenic micronuclei removed by autophagy. Hum Mol Genet 26: 3960–3972 29016854 10.1093/hmg/ddx283

[167] Hatch EM , Fischer AH , Deerinck TJ , Hetzer MW (2013) Catastrophic nuclear envelope collapse in cancer cell micronuclei. Cell 154: 47–60 23827674 10.1016/j.cell.2013.06.007 PMC3749778

[168] Dou Z , Ghosh K , Vizioli MG , Zhu J , Sen P , Wangensteen KJ , Simithy J , Lan Y , Lin Y , Zhou Z *et al* (2017) Cytoplasmic chromatin triggers inflammation in senescence and cancer. Nature 550: 402–406 28976970 10.1038/nature24050 PMC5850938

[169] Yang H , Wang H , Ren J , Chen Q , Chen ZJ (2017) cGAS is essential for cellular senescence. Proc Natl Acad Sci USA 114: E4612–E4620 28533362 10.1073/pnas.1705499114 PMC5468617

[170] Gluck S , Guey B , Gulen MF , Wolter K , Kang TW , Schmacke NA , Bridgeman A , Rehwinkel J , Zender L , Ablasser A (2017) Innate immune sensing of cytosolic chromatin fragments through cGAS promotes senescence. Nat Cell Biol 19: 1061–1070 28759028 10.1038/ncb3586 PMC5826565

[171] Chattopadhyay S , Marques JT , Yamashita M , Peters KL , Smith K , Desai A , Williams BR , Sen GC (2010) Viral apoptosis is induced by IRF‐3‐mediated activation of Bax. EMBO J 29: 1762–1773 20360684 10.1038/emboj.2010.50 PMC2876960

[172] Czabotar PE , Westphal D , Dewson G , Ma S , Hockings C , Fairlie WD , Lee EF , Yao S , Robin AY , Smith BJ *et al* (2013) Bax crystal structures reveal how BH3 domains activate Bax and nucleate its oligomerization to induce apoptosis. Cell 152: 519–531 23374347 10.1016/j.cell.2012.12.031

[173] Gulen MF , Koch U , Haag SM , Schuler F , Apetoh L , Villunger A , Radtke F , Ablasser A (2017) Signalling strength determines proapoptotic functions of STING. Nat Commun 8: 427 28874664 10.1038/s41467-017-00573-w PMC5585373

[174] Tang CH , Zundell JA , Ranatunga S , Lin C , Nefedova Y , Del Valle JR , Hu CC (2016) Agonist‐mediated activation of STING induces apoptosis in malignant B cells. Can Res 76: 2137–2152 10.1158/0008-5472.CAN-15-1885 PMC4873432 26951929

[175] Davoli T , Uno H , Wooten EC , Elledge SJ (2017) Tumor aneuploidy correlates with markers of immune evasion and with reduced response to immunotherapy. Science 355: eaaf8399 28104840 10.1126/science.aaf8399 PMC5592794

[176] Senovilla L , Vitale I , Martins I , Tailler M , Pailleret C , Michaud M , Galluzzi L , Adjemian S , Kepp O , Niso‐Santano M *et al* (2012) An immunosurveillance mechanism controls cancer cell ploidy. Science 337: 1678–1684 23019653 10.1126/science.1224922

[177] Giampazolias E , Zunino B , Dhayade S , Bock F , Cloix C , Cao K , Roca A , Lopez J , Ichim G , Proics E *et al* (2017) Mitochondrial permeabilization engages NF‐kappaB‐dependent anti‐tumour activity under caspase deficiency. Nat Cell Biol 19: 1116–1129 28846096 10.1038/ncb3596 PMC5624512

[178] Barboule N , Truchet I , Valette A (2005) Localization of phosphorylated forms of Bcl‐2 in mitosis: co‐localization with Ki‐67 and nucleolin in nuclear structures and on mitotic chromosomes. Cell Cycle 4: 590–596 15876860 10.4161/cc.4.4.1588

[179] Sakurikar N , Eichhorn JM , Chambers TC (2012) Cyclin‐dependent kinase‐1 (Cdk1)/cyclin B1 dictates cell fate after mitotic arrest via phosphoregulation of antiapoptotic Bcl‐2 proteins. J Biol Chem 287: 39193–39204 22965228 10.1074/jbc.M112.391854 PMC3493959

[180] Basu A , Haldar S (2003) Identification of a novel Bcl‐xL phosphorylation site regulating the sensitivity of taxol‐ or 2‐methoxyestradiol‐induced apoptosis. FEBS Lett 538: 41–47 12633850 10.1016/s0014-5793(03)00131-5

[181] Wang J , Beauchemin M , Bertrand R (2012) Phospho‐Bcl‐x(L)(Ser62) plays a key role at DNA damage‐induced G(2) checkpoint. Cell Cycle 11: 2159–2169 22617334 10.4161/cc.20672 PMC3368867

[182] Kobayashi S , Lee SH , Meng XW , Mott JL , Bronk SF , Werneburg NW , Craig RW , Kaufmann SH , Gores GJ (2007) Serine 64 phosphorylation enhances the antiapoptotic function of Mcl‐1. J Biol Chem 282: 18407–18417 17463001 10.1074/jbc.M610010200

